# Patients with idiopathic recurrent miscarriage have abnormally high TGFß+ blood NK, NKT and T cells in the presence of abnormally low TGFß plasma levels

**DOI:** 10.1186/s12865-019-0290-3

**Published:** 2019-03-04

**Authors:** Li Zhu, Mostafa Aly, Ruben Jeremias Kuon, Bettina Toth, Haihao Wang, Hristos Karakizlis, Rolf Weimer, Christian Morath, Eman Ibrahim, Naruemol Ekpoom, Gerhard Opelz, Volker Daniel

**Affiliations:** 1 Department of Hematology, Tongji Hospital, Huazhong University of Science and Technology, Wuhan, 430030 China; 2 Transplantation Immunology, Institute of Immunology, University Hospital Heidelberg, Im Neuenheimer Feld 305, 69120 Heidelberg, Germany; 3 Department of Nephrology, University of Heidelberg, Im Neuenheimer Feld 162, Heidelberg, Germany; 4 Nephrology unit, Internal Medicine Department, Assiut University, Assiut, Egypt; 5 Department of Obstetrics and Gynecology, University Hospital Heidelberg, Im Neuenheimer Feld 440, 69120 Heidelberg, Germany; 6 Department of Gynecological Endocrinology and Reproductive Medicine, Medical University Innsbruck, Anichstrasse 35, 6020 Innsbruck, Austria; 7 Department of Surgery, Tongji Hospital, Huazhong University of Science and Technology, Wuhan, 430030 China; 8 Department of Internal Medicine, University of Giessen, Klinikstrasse 33, D-35385 Giessen, Germany

**Keywords:** Idiopathic recurrent miscarriage, TGFß, IL4, IL10, TNFα, NK cells, Plasma cytokines, Cell cultures, K562, Supernatants, Patients, Transplantation

## Abstract

**Background:**

Previously, we demonstrated up-regulated activated CD4+ and CD8+ T lymphocytes as well as up-regulated cytotoxic NK cells in the blood of patients with idiopathic recurrent miscarriage. In the present study, we tried to identify deficiencies in counter-regulating immune mechanisms of these patients.

**Method:**

Cytokines were determined in NK cells and in plasma samples of 35 healthy controls, 33 patients with idiopathic recurrent miscarriage, 34 patients with end stage renal disease, 10 transplant patients early and 37 transplant patients late post-transplant using flow-cytometry and luminex. In addition, cytokines were studied in supernatants of cell cultures with peripheral blood mononuclear cells stimulated in-vitro with tumor cell line K562.

**Results:**

Patients with idiopathic recurrent miscarriage exhibited the highest absolute cell counts of circulating TGFß1+ NK, NKT and T lymphocytes and the lowest TGFß1 plasma levels of all study groups (for all *p* < 0.050). In-vitro, peripheral blood mononuclear cells of patients with idiopathic recurrent miscarriage showed high spontaneous TGFß1 production that could not be further increased by stimulation with K562, indicating increased consumption of TGFß1 by activated cells in the cell culture. Moreover, patients with idiopathic recurrent miscarriage had abnormally high IL4+ as well as abnormally high IFNy+ NK cells (*p* < 0.010) but similar IL10+ NK cell numbers as female healthy controls and showed the lowest plasma levels of IL10, TGFß3, IL1RA, IL1ß, IL5, IL6, IL8, IL17, TNFα, GM-CSF, TPO and VEGF and the highest plasma levels of G-CSF, FGF-basic, CCL3 and CXCL5 as compared to female HC and female transplant recipients (for all p < 0.050).

**Conclusions:**

Patients with idiopathic recurrent miscarriage show an activated immune system that can hardly be stimulated further and cannot be efficiently down-regulated by up-regulated TGFß1+ and IL4+ NK, NKT and T lymphocytes which are present concomitantly in these patients. The strongly decreased TGFß and IL10 plasma levels indicate deficient down-regulation and reflect a dysbalance of the immune system in patients with idiopathic recurrent miscarriage. These findings may be relevant for explaining the pathogenesis of idiopathic recurrent miscarriage.

**Electronic supplementary material:**

The online version of this article (10.1186/s12865-019-0290-3) contains supplementary material, which is available to authorized users.

## Background

Recurrent loss of pregnancy is an important reproductive health issue, affecting 2–5% of couples [1]. Almost half of the cases remain unexplained and are treated empirically, using progesterone supplementation, anticoagulation, and/or immunomodulatory approaches [1]. Immunological causes, such as immunological rejection or development of an intrauterine micromilieu that is harmful for fetus and pregnancy, are suspected reasons for idiopathic recurrent miscarriage (iRM) [2, 3]. In previous studies, we were able to show highly elevated uterine NK cells in iRM compared to non-iRM patients [4]. Furthermore, compared with female healthy controls (HC), iRM patients showed higher counts of circulating activated CD4+ and CD8+ T cells [5] and cytotoxic NK cells [6] and lower levels of circulating presumably immunoregulatory IL10 + CD56bright NK cells [6]. In contrast, renal transplant recipients with functioning grafts late post-transplant exhibited lower cytotoxic and higher IL10 + CD56bright NK cell counts than HC [6]. Moreover, in transplant recipients with good long-term graft outcome, high cytotoxic NK cells were associated with impaired graft outcome whereas high immunoregulatory IL10 + CD56bright NK cells were associated with good long-term graft function. We speculate that these findings on cytotoxic and immunoregulatory IL10 + CD56bright NK cells might have relevance for the fetal allograft in pregnant women. Because women with idiopathic RM showed high cytotoxic and low immunoregulatory IL10 + CD56bright NK cells in the circulation, we concluded that the immune system of iRM patients is dysbalanced and that this might favor immunologically-induced abortion. Mor et al. postulated that the first trimester of pregnancy is associated with inflammation, which is required for blastocyst implantation [3]. The second trimester is characterized by an anti-inflammatory and T helper 2 (TH2)-type immune microenvironment that is necessary for fetal growth, and in the third trimester there is a switch to an inflammatory and TH1-type immune state, which is necessary for labor and delivery [3]. We hypothesize that iRM patients exhibit a continued inflammatory immune response during the second trimester that cannot be counter-regulated by increasing inhibitory immune mechanisms, which eventually results in an unfavorable intrauterine micromilieu that is harmful for the fetus and results in the loss of pregnancy.

We compared iRM patients with female HC as well as a series of HC including males to identify abnormally increased or decreased cell subsets and cytokine levels in-vivo and in-vitro. Additionally, iRM patients were compared with dialysis patients and transplant recipients late post-transplant. All transplant recipients received low dose immunosuppression and were free of infection or rejection. As shown in our previous publication, this group consists of patients with down-regulated cytotoxic effector mechanisms and up-regulated counter-regulating immunoregulatory mechanisms forming an immune system in balance [7]. While immunosuppressive drugs inhibit the formation of cytokines and cytokine receptors, cells that already express certain cytokines and cytokine receptors act similar in patients with or without immunosuppressive drug treatment. We hypothesize that the comparison of iRM patients with kidney recipients late post-transplant represents a comparison of patients with an unbalanced to patients with a balanced immune system. Immune mechanisms that are associated with good or poor graft outcome in transplant recipients might also be associated with successful or unsuccessful pregnancy of the semi-allogeneic fetus.

In the present study, we looked for indications for a dysregulation of IL4, IL10 and TGFß induced counter-regulating inhibitory immune mechanisms. We investigated IL4+, IL10+, TGFß+ and IFNy+ NK, NKT and T cells in the blood of HC, iRM and transplant patients and compared intracellular cytokine production with cytokine levels measured in plasma. In addition, we stimulated NK cells in PBMC cultures using the tumor cell line K562 as stimulator and measured cytokine expression in NK cells before and after stimulation and in the supernatants of the in-vitro stimulated cells. We speculated that cells with T cell receptor interact with HLA on allogeneic cells whereas NK cells without T cell receptor might react with cells missing or expressing particular HLA class I antigens (inhibiting or activating KIRs) or cells expressing stress-induced MICA and MICB (ligand NKG2D) and/or HLA-E (ligand NKG2A) on the cell surface [8].

## Results

**Fig. 1:**
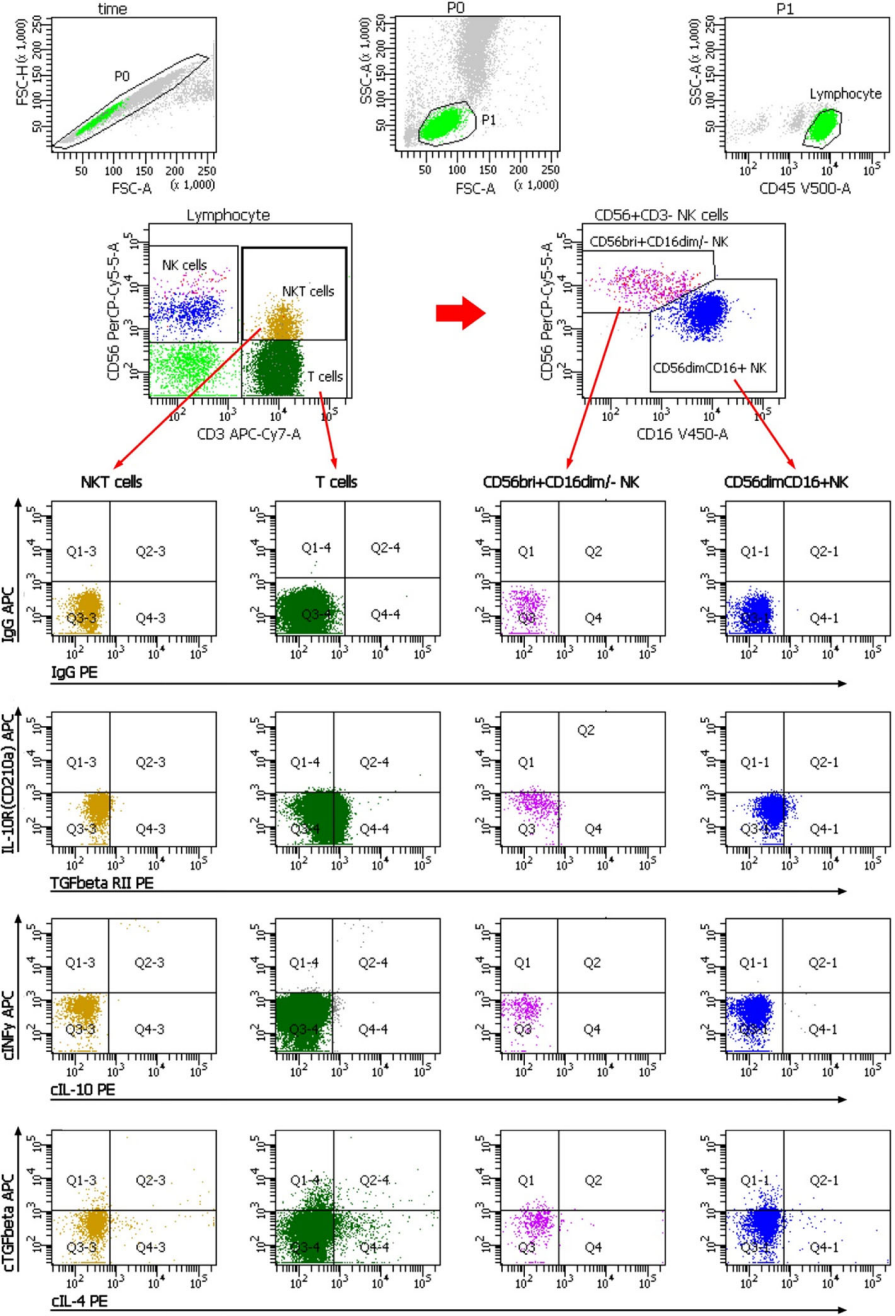
Gating strategy for the determination of NK, NKT and T cell subsets. After excluding doublets from the total of acquired events, peripheral blood lymphocytes (PBL) were gated according to FSC/SSC and CD45/SSC dot plot. Then, CD3-CD56+ NK cells, CD3 + CD56+ NKT cells and CD3+ T cells were gated in the CD3-APC/CD56-PerCPCy5.5 dot plot. CD3-CD56+ NK cells were further analyzed according to the intensity of the CD56 and CD16 expression (CD16-V450/CD56-PerCPCy5.5 dot plot). Further, dependent on isotype controls, subsets of NKT cells, T cells, CD56brightCD16dim/− NK cells, CD56dimCD16+ NK cells and CD56 + HLADR+ NK cells were analyzed using the depicted gate settings in dot plots of IL10R/TGFßRII, cIFNy/cIL10, cTGFß/cIL4. FSC, forward-scattered light, SSC, side-scattered light

We studied 33 iRM patients, 35 HC (19 female), 34 ESRD patients (15 female), 10 renal transplant recipients early (3 female) and 37 renal transplant recipients late (14 female) post-transplant. Results of females are detailed in the text whereas findings of both males and females are depicted in the figures of the Additional files 1, 2, and 3. Gating strategy is shown in Fig. 1.

### Intracellular cytokine production of blood NK, NKT and T cells

**Fig. 2:**
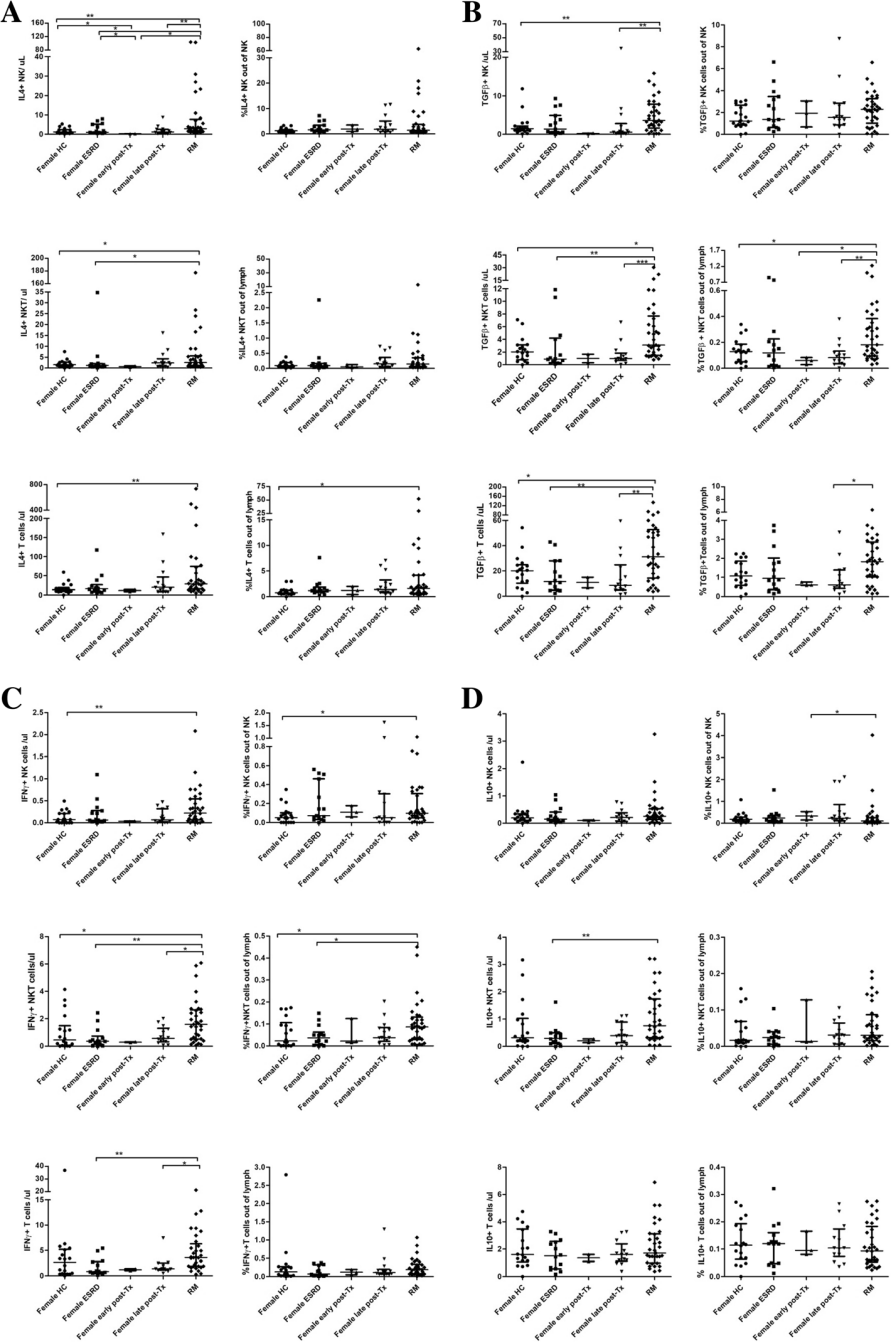
a+b IL4+, TGFß1+, IL10+ and IFNy+ NK, NKT and T cell counts in peripheral blood. iRM patients showed higher absolute numbers of NK, NKT and T cells producing IL4 and TGFß1 than female HC, higher absolute numbers of IFNy+ NK and NKT cells than female HC and higher IL4+ and TGFß1+ NK and TGFß1+ and IFNy+ NKT and T cells than female kidney transplant recipients late post-transplant (for all *p* < 0.050) whereas IL10+ NK cells were similar in all transplant patient groups and female HC (**a**, **b**, **c** and **d**). Similar trends were observed for the proportions of IL4+ T cells, TGFß1+ NKT cells and IFNy+ NK and NKT lymphocytes (*p* < 0.050). Nineteen female HC, 15 female ESRD, 14 female renal transplant recipients late and 3 early post-transplant and 33 iRM patients were studied. Data are given as median ± interquartile range

iRM patients showed higher absolute numbers of circulating NK, NKT and T lymphocytes producing IL4 and TGFß1 and higher absolute numbers of NK and NKT cells with IFNy production than female HC (for all *p* < 0.050) (Fig. 2 a +b). Moreover, iRM patients had higher absolute counts of IL4+ NK and NKT cells than female ESRD patients (*p* < 0.050), higher TGFß+ NK cells than female patients late post-transplant and higher TGFß+ and IFNy+ NKT and T cells than female ESRD patients and female transplant recipients late post-transplant (for all *p* < 0.050) (Fig. 2 a+b). IL10+ NK, NKT and T cells were similar in all groups with the exception of higher absolute IL10+ NK cell counts in female transplant recipients early post-transplant and higher IL10+ NKT cells in female ESRD patients compared to iRM patients (for all *p* < 0.050) (Fig. 2 a+b).

When, in addition, iRM patients were compared with male and female HC and male and female ESRD and transplant patients (Additional file 1: Figure S1a + b), iRM patients showed higher absolute counts of IL4+, TGFß+ and IFNy+ NK, NKT and T cells than male and female HC, ESRD patients and patients late post-transplant (p < 0.050). Moreover, iRM patients had higher absolute numbers of IL10+ NKT cells than male and female ESRD patients and male and female kidney transplant recipients early and late post-transplant (for all *p* < 0.050) (Additional file 1: Figure S1a + b). When, in addition, relative cell numbers were analyzed, iRM patients showed significantly higher proportions of IL4+, TGFß+ and IFNy+ NK, NKT and T cells and of IL10+ NK cells than male and female HC (for all *p* < 0.050) (Additional file 1: Figure S1a + b). The similar trends of relative and absolute cell numbers suggest a strong up-regulation of cell subsets with immunosuppressive (IL4 and TGFß) as well as immunostimulatory (IFNy) phenotype in iRM patients compared to female HC and female transplant recipients.

### Cytokine receptors on blood NK, NKT and T cells

**Fig. 3:**
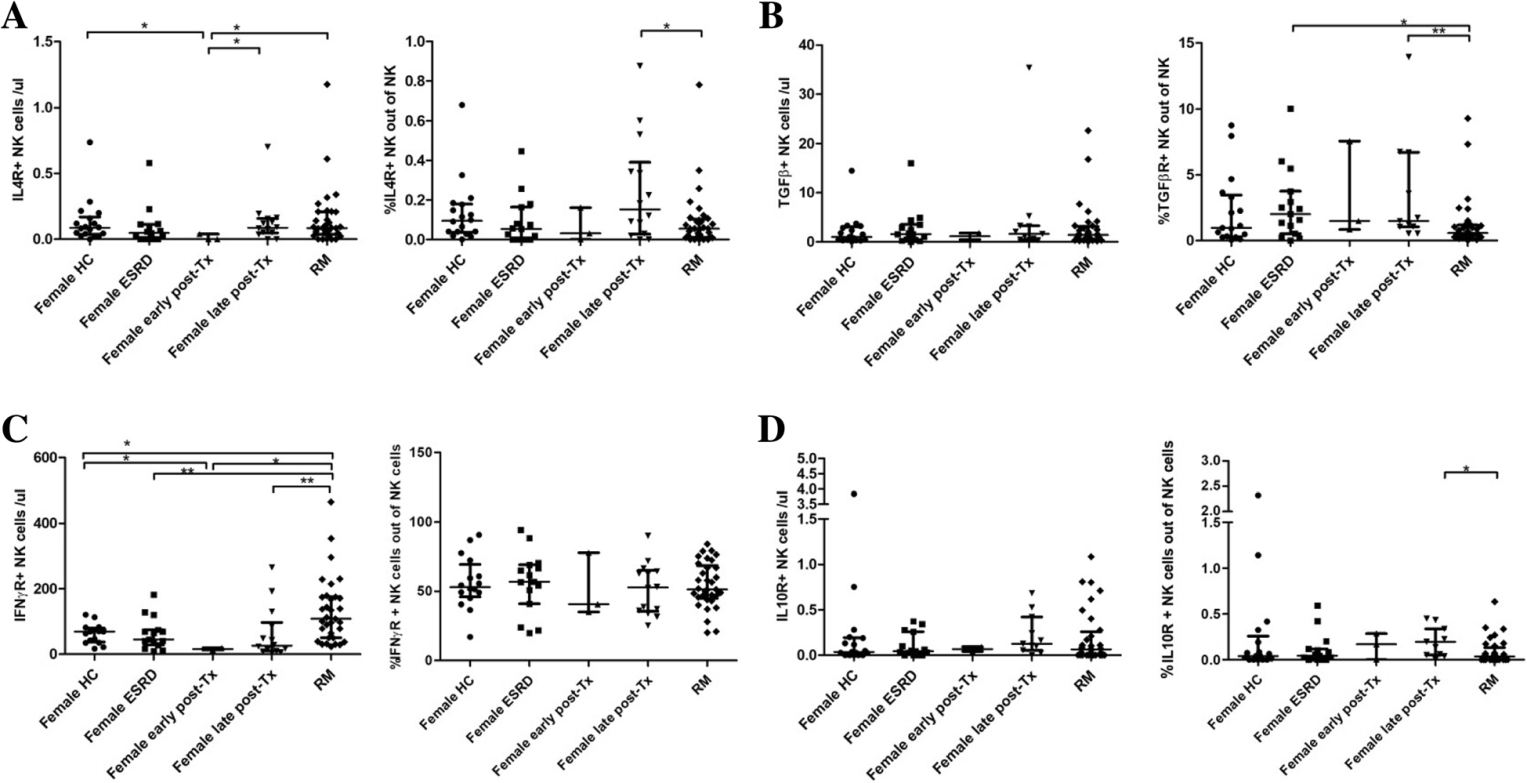
**a**-**d** IL4R+, TGFßR+, IFNyR+ and IL10R+ NK cells in peripheral blood. iRM patients had lower relative counts of IL4R+, TGFßR+ and IL10R+ and higher absolute numbers of IFNyR+ NK cells than female kidney graft recipients late post-transplant (for all *p* < 0.050). Moreover, iRM patients showed the highest absolute counts of IFNyR+ NK cells of all examined groups (for all p < 0.050). The data suggest a lower expression of IL4R, TGFßR and IL10R on NK cells of iRM patients but an increased response of IFNyR+ NK cells compared to female transplant recipients. Nineteen female HC, 15 female ESRD, 14 female renal transplant recipients late and 3 early post-transplant and 33 iRM patients were studied. Data are given as median ± interquartile range

An up-regulation of cytokine receptors, measured as relative increase of cytokine receptor positive cells, signals a strong need for paracrine produced cytokines. NK cells of iRM patients showed lower expression of IL4R, TGFßR and IL10R (for all p < 0.050) but similar expression of IFNyR compared to NK cells of female transplant recipients late post-transplant. Conversely, iRM patients had the highest absolute counts of IFNyR+ NK cells among all female patients and female HC (p < 0.050) while absolute cell counts of IL4R+, TGFßR+ and IL10R+ NK cells were similar in RM patients, female HC, female ESRD patients and female transplant recipients late post-transplant (for all *p* > 0.050). The data suggest a low need for paracrine produced IL4, IL10 and TGFß and a strong need and consumption of auto- and paracrine produced IFNy in RM patients (Fig. 3) (Additional file 2: Figure S2) to maintain the strong IFNy-dependent cytotoxic potential in RM patients, as published previously [6].

### Cytokine levels in plasma

**Fig. 4:**
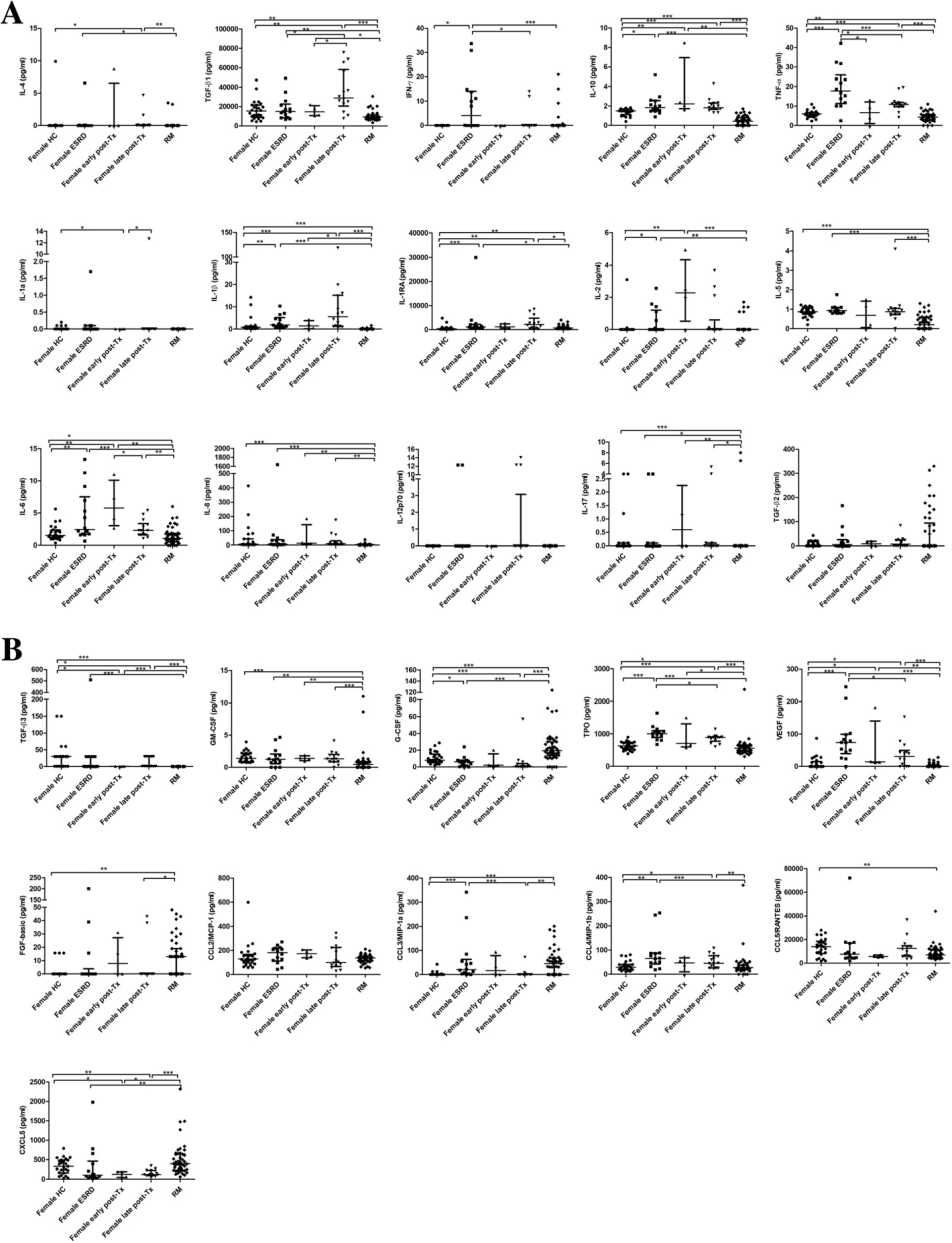
**a**+**b** Plasma levels of cytokines and chemokines. iRM patients showed the lowest plasma levels of the pro-inflammatory/ inflammatory cytokines TNFα, IL1ß, IL6, IL17 and GM-CSF, of anti-inflammatory IL1RA, IL10, TGFß1 and TGFß3 and of the cytokines IL5, IL8, VEGF and TPO compared to female HC and female transplant recipients late post-transplant (for all *p* < 0.050). They had also lower levels of the chemokines CCL5/RANTES than female HC and of CCL4/MIP1ß than female ESRD and female transplant recipients late post-transplant (for all p < 0.050). Conversely, iRM patients showed the highest plasma levels of G-CSF, FGF-basic and CCL3/MIP1α as well as very high plasma levels of CXCL5 compared to female HC and female transplant recipients late post-transplant (for all p < 0.050). Interestingly, plasma IFNy, TGFß2, IL1α, IL2, IL12 and CCL2/MCP-1 were similar in iRM patients, female HC and female transplant recipients late post-transplant (*p* > 0.050). Twenty-six female HC, 15 female ESRD, 4 female renal transplant recipients early and 15 female renal transplant recipients late post-transplant as well as 33 iRM patients were studied. Data are given as median ± interquartile range

Plasma levels of IL4, TGFß1, IFNy and IL10 in the different patient groups were inverse to absolute counts of IL4+, TGFß1+, IFNy+ and IL10+ cell subsets in the peripheral blood. Plasma levels were lowest in iRM patients but highest in female transplant recipients (Fig. 4 a+b). iRM patients showed the lowest TGFß1 and IL10 plasma levels when compared to female HC, female ESRD patients and female transplant recipients early and late post-transplant, lower IL4 plasma levels than female transplant recipients late post-transplant and female ESRD patients and lower plasma IFNy than female ESRD patients (for all *p* < 0.050) (Fig. 4 a+b). In contrast, female transplant recipients late post-transplant showed higher IL4, TGFß1 and IL10 (for all p < 0.050) but similar IFNy plasma levels as female HC (*p* > 0.050). In addition, iRM patients showed the lowest plasma levels of the pro-inflammatory/ inflammatory cytokines TNFα, IL1ß, IL6, IL17 and GM-CSF, of anti-inflammatory IL-1RA and TGFß3 and of the cytokines IL5, IL8, TPO and VEGF compared to female HC and female transplant recipients late post-transplant (for all p < 0.050) (Fig. 4 a+b). They had also lower levels of the chemokines CCL5/RANTES than female HC and of CCL4/MIP1ß than female ESRD and female transplant recipients late post-transplant (for all p < 0.050) (Fig. 4 a+b). Conversely, iRM patients showed the highest plasma levels of G-CSF, FGF-basic, CCL3/MIP1α as well as CXCL5 compared to female HC and female transplant recipients late post-transplant (for all p < 0.050) (Fig. 4 a+b). Interestingly, plasma IFNy, TGFß2, IL1α, IL2, IL12 and CCL2/MCP-1 were similar in iRM patients, female HC and female transplant recipients late post-transplant (p > 0.050) (Fig. 4 a+b). Similar results were observed when plasma cytokine levels of iRM patients were compared with those of male and female transplant patients and HC (Additional file 3 Figure S3a+b). In summary, the data show abnormally low plasma IL10, TGFß1, TNFα, IL1ß, IL1RA, TPO and VEGF in iRM patients and abnormally high levels of these cytokines, and in addition of IL4, in female transplant recipients late post-transplant. The data suggest a systemic down-regulation of anti-inflammatory cytokines in iRM patients and a systemic up-regulation of these cytokines in female transplant recipients late post-transplant.

Because iRM patients showed abnormally high numbers of blood cells producing anti-inflammatory cytokines in the presence of abnormally low plasma levels of these cytokines, we studied in cell cultures with PBMC whether the production of anti-inflammatory cytokines can be increased in NK cells using K562 cells as a NK cell-specific stimulus, whether the stimulated NK cells were able to release these cytokines in the supernatant of the cell culture and whether consumption of the secreted cytokines (by activated NK cells) is increased in-vitro.

### IL4, TGFß1, IL10 and IFNy in NK cells after stimulation with K652 in-vitro

**Fig. 5:**
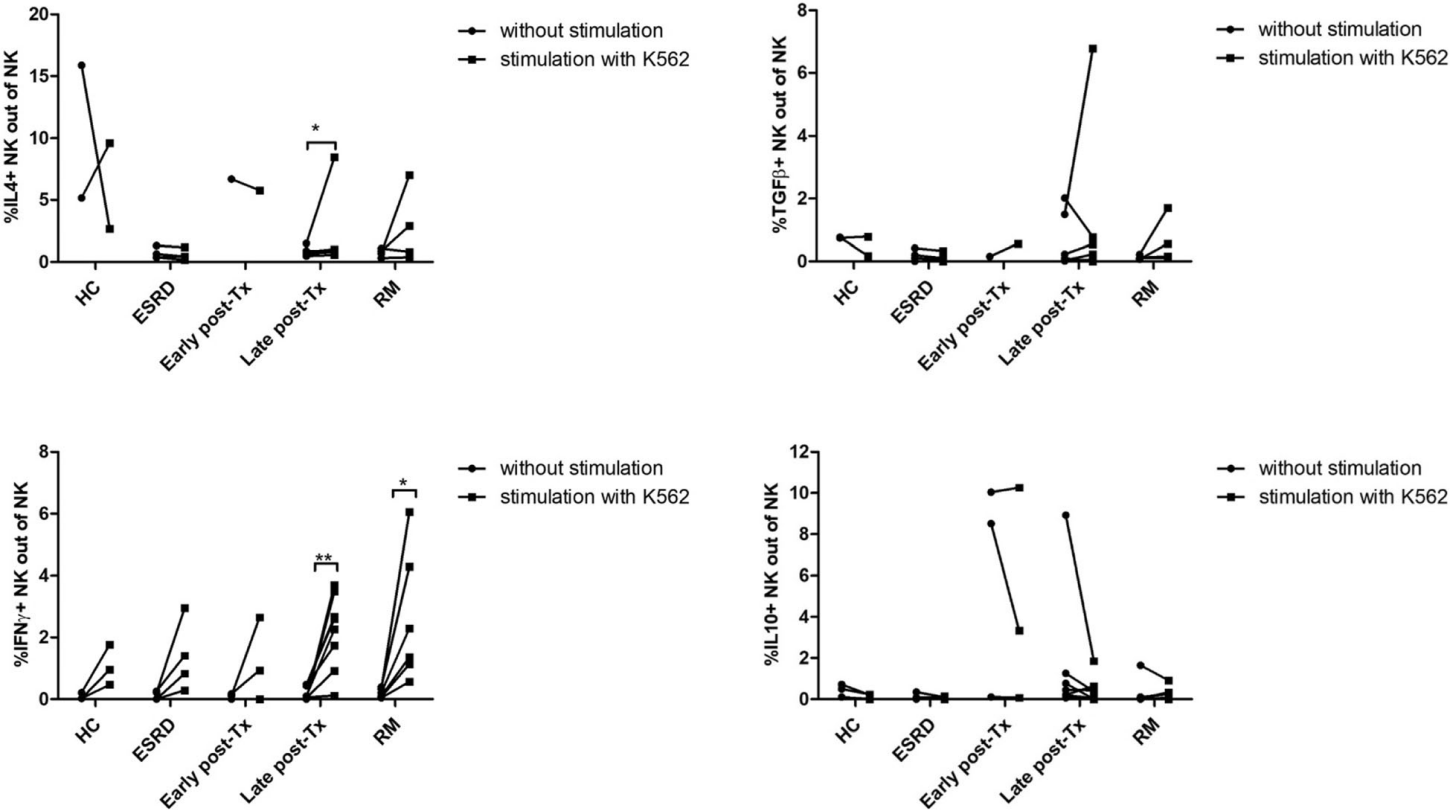
IL4+, TGFß1+, IL10+ and IFNy+ NK cells before and after stimulation with K562 in-vitro. PBMC of iRM patients, kidney transplant recipients > 3 months post-transplant, kidney transplant recipients < 3 months post-transplant, ESRD patients and HC were incubated with tumor cell line K562 using an E:T ratio of 5:1. K562 stimulates particularly NK cells. Proportions of IL4+, TGFß1+, IFNy+ and IL10+ NK cells were measured before and after stimulation in the cell cultures. iRM patients and kidney transplant recipients > 3 months post-transplant showed an increase of IFNy+ NK cells after stimulation, the latter, in addition, an increase of IL4+ NK cells (for all p < 0.050). Number of patients tested for IFNy+ and IL10+ and in brackets IL4+ and TGFß1+ NK cells: 3 (2) HC, 4 (4) ESRD, 3 (1) early and 8 (6) late post-transplant and 6 (4) iRM patients

PBMC of 6 iRM patients, 8 kidney transplant recipients > 3 months post-transplant, 3 kidney transplant recipients < 3 months post-transplant, 4 ESRD patients and 3 HC were incubated with tumor cell line K562 using an E:T ratio of 5:1. K562 stimulates particularly NK cells and proportions of IL4+, TGFß1+, IL10+ and IFNy+ NK cells were measured before and after stimulation in the cell cultures. iRM patients and kidney transplant recipients > 3 months post-transplant showed an increase of IFNy+ NK cells after stimulation, and the latter, in addition, an increase of IL4+ NK cells compared to female HC (for all *p* < 0.050) (Fig. 5) suggesting that in iRM and transplant patients IFNy production of NK cells can be further increased by stimulation whereas production of TGFß1 and IL10 appears to remain stable.

### IL4, TGFß1, IL10 and IFNy in supernatants after stimulation with K562 in-vitro

**Fig. 6:**
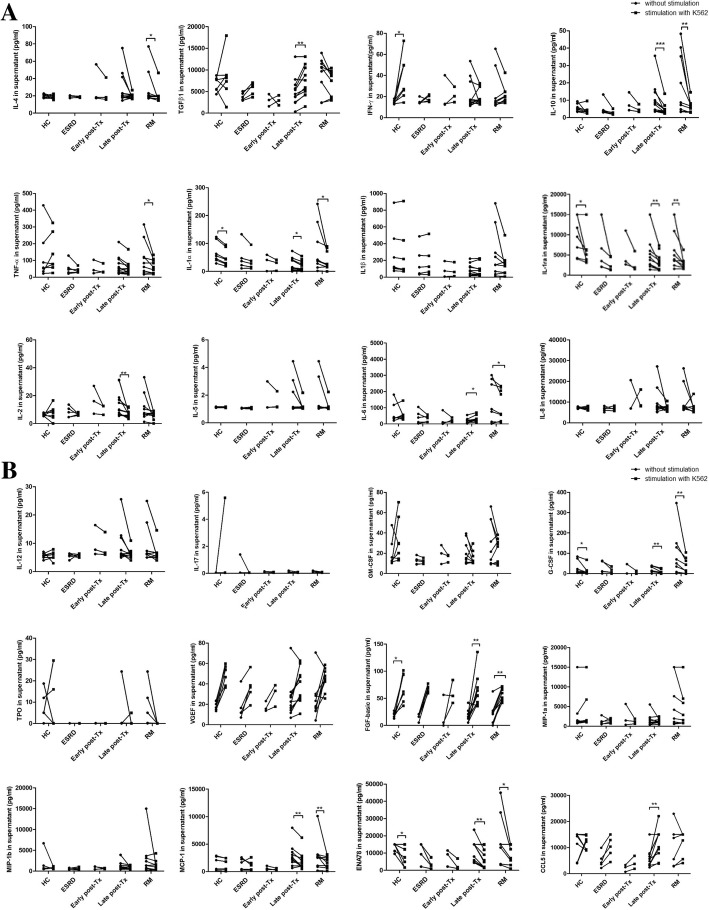
**a** + **b** Cytokine and chemokine levels in cell culture supernatants. PBMC of 9 iRM patients, 11 kidney transplant recipients > 3 months post-transplant, 3 kidney transplant recipients < 3 months post-transplant, 5 ESRD patients and 7 HC were incubated with tumor cell line K562 using an E:T ratio of 5:1. K562 stimulates particularly NK cells. Cytokine and chemokine levels were measured in the supernatants of unstimulated and stimulated cell cultures. The most interesting finding was that cells of iRM patients showed the highest spontaneous TGFß1 production of all female patients and HC after incubation in medium indicating a strong pre-activation of cells (iRM vs female transplant recipients late post-transplant *p* = 0.080). After stimulation, TGFß1 levels were lower than before stimulation indicating a strong consumption of TGFß1 by activated NK cells of iRM patients. In contrast, transplant recipients late post-transplant exhibited a higher TGFß1 production after than before stimulation (p < 0.050). A similar high spontaneous production and strong consumption of cytokines by activated cells was observed for IL6 in the supernatants of iRM patients (iRM vs female transplant recipients late post-transplant *p* = 0.068)

In-vitro studies were performed with cell suspensions of 7 HC, 9 iRM and 5 ESRD patients as well as 3 patients early and 11 late post-transplant. NK cells were stimulated in PBMC cultures with K562 using an E:T ratio of 5:1. IL4, TGFß1, IL10 and IFNy levels were analyzed in supernatants obtained with and without stimulation. The most interesting finding was that cells of iRM patients after incubation in medium showed the highest spontaneous TGFß1 production of all female patients and HC (vs female transplant recipients late post-transplant *p* = 0.080). After stimulation, there was a trend that TGFß1 levels in the supernatants of iRM patients were lower than before stimulation indicating a strong consumption of TGFß1 by activated NK cells. In contrast, transplant recipients late post-transplant exhibited TGFß1 levels in the supernatants that increased during stimulation (*p* < 0.050) (Fig. 6 a+b). A similar high spontaneous cytokine production and strong cytokine consumption by activated cells was observed for IL6 in the supernatants of iRM patients (iRM vs female transplant recipients late post-transplant *p* = 0.068). Similar to TGFß, IL6 levels decreased during stimulation in cell cultures of iRM patients but increased in those of transplant recipients late post-transplant (for all p < 0.050). Other cytokines in the supernatants showed similar production and consumption patterns in iRM patients, transplant recipients as well as HC (Fig. 6 a+b). The data suggest that iRM patients have pre-activated NK cells with strong spontaneous TGFß1 production in the circulation that strongly consume TGFß1. The strong TGFß1 consumption might contribute to the low TGFß1 plasma levels in these patients.

## Discussion

In a previous study we observed that, compared with female healthy controls, iRM patients possessed abnormally high cytotoxic and abnormally low IL10 + CD56bright NK cells in the peripheral blood, whereas renal transplant recipients on low-dose immunosuppression late post-transplant exhibited abnormally low cytotoxic and abnormally high IL10 + CD56bright NK cell counts [6]. Moreover, iRM patients showed higher absolute numbers of NK cells with predominantly inhibitory killer-cell immunoglobulin-like receptors (KIR) and CD94/NKG2 receptors than female HC [8]. They exhibited significantly increased levels of NK cells with inhibitory CD158a, CD158b, CD158e, NKG2A and stimulatory NKG2D receptors, whereas female ESRD patients had normal and female transplant recipients lower or normal numbers of these cells compared to female HC [8]. Interestingly, the phenotype CD158a+/CD158b+/CD158e+/NKG2A+/NKG2D+ was more common in iRM patients than in female HC and female transplant recipients late post-transplant, supporting the hypothesis that iRM patients have an abnormal up-regulation of inhibitory KIR and NKG2A receptors as well as of stimulatory NKG2D determinants [8]. We concluded that the dysbalance of cytotoxic and potentially immunoregulatory NK cells in iRM patients might contribute to the pathogenesis of iRM and that the balance of low cytotoxic and high IL10 + CD56bright NK cells in renal transplant recipients might be favorable for good long-term graft acceptance. In the present study, we investigated additional NK cell subsets with presumable immunoregulatory function in the blood of iRM and transplant patients as well as HC, and we analyzed whether these cells might affect the cytokine levels in plasma and contribute to a systemic immunosuppressive cytokine pattern.

Our data show that iRM patients have abnormally high absolute cell counts of IL4+ and TGFß1+ NK, NKT and T lymphocytes in the blood that show low IL4R and TGFßR expression. These cells do not need paracrine secreted IL4 and TGFß1 because of their own strong production of these cytokines. Transplant recipients, in contrast, have normal cell counts of IL4+ and TGFß1+ NK, NKT and T lymphocytes and abnormally high IL4 and TGFß1 plasma levels. Although iRM patients show the highest numbers of IL4+ and TGFß1+ cell subsets in the blood, they have the lowest levels of these cytokines in plasma compared to HC and all patient groups. The data suggest that NK, NKT and T lymphocyte counts do not strongly correlate with plasma levels of IL4 and TGFß1 and do not induce a systemic immunosuppressive milieu in the circulation. Presumably, the peripherally increased IL4+ and TGFß+ NK, NKT and T cells in iRM patients originate from the uterus, however, they are unable to establish a micromilieu in the uterus that is protective for the fetus [9, 10].

The opposite holds true for IL10. In the present study, summarizing IL10 + CD56bright and IL10 + CD56dimCD16+ NK cells as IL10+ NK cells, there was no difference in IL10+ NK cell numbers among iRM patients, female HC and female transplant recipients. In a previous study, we reported on abnormally high IL10 + CD56bright NK cells in transplant recipients and abnormally low IL10 + CD56bright NK cells in iRM patients [6] and these observations are paralleled by abnormally low IL10 plasma levels in iRM patients and abnormally high IL10 plasma levels in transplant recipients in the present study. The data support the hypothesis that plasma IL10 originates in part from IL10 + CD56bright NK cells and that IL10 + CD56bright NK cells might strongly contribute to systemic immunosuppression in the circulation. Moreover, in the previous study we described a striking association of IL10 + CD56bright NK cell counts in the blood with graft function in renal transplant recipients [6] suggesting that this NK cell subset might be strongly immunoregulatory. In the same study, CD8+ perforin+granzymeB+ NK cells, which can be thought of as the opponent of IL10 + CD56bright NK cells, were shown to be associated with impaired graft outcome [6]. The combination of high IL10 + CD56bright NK cell counts and high IL10 plasma levels in transplant recipients with good long-term graft function supports the hypothesis that IL10+ NK cell subsets might have a strong immunoregulatory role in transplant recipients. IL10+ NK cells that suppressed antigen-specific T-cell responses were reported by Deniz et al. [11] and others as summarized by Vivier et al. [12]. iRM patients, in contrast, show a deficit of this presumably immunoregulatory subset in the circulation and we assume that this deficit might contribute to the pathogenesis of idiopathic RM. As summarized by Mor et al. [3], a pro-inflammatory immune response supports implantation and placentation of the blastocyte and initiates pregnancy, followed by an anti-inflammatory stage of fetal growth and a final pro-inflammatory switch for labor and delivery. Our data suggest that patients with idiopathic RM do not switch to an anti-inflammatory stage.

In a previous study, we reported on abnormally high activated CD3 + DR+, CD4 + DR+ and CD8 + DR+ T lymphocytes in the circulation of patients with idiopathic RM [5]. When these pre-activated T lymphocytes were stimulated in-vitro using phytohaemagglutinin, pokeweed mitogen or anti-CD3 monoclonal antibody, they showed decreased in-vitro proliferation compared to lymphocytes of healthy female controls tested in parallel [5]. Similar results were obtained in the present study for the production of TGFß1 in NK cells. Separated peripheral blood lymphocytes of iRM patients showed the highest spontaneous TGFß1 production of all study groups as determined in cell culture supernatants. When NK cells were stimulated using tumor cell line K562, TGFß1 levels in supernatants were lower after stimulation than prior to stimulation, suggesting strong spontaneous production of TGFß1 in circulating NK cells that could not be increased by additional stimulation in-vitro and decreased due to consumption by activated cells.

After abortion, patients with idiopathic RM have a combination of abnormally high proportions of circulating pre-activated T lymphocytes, high cytotoxic NK cells as well as high IL4+, TGFß1+ and IFNy+ NK cells and, in addition, low proportions of circulating IL10 + CD56bright NK cells, low plasma levels of IL4, IL10, TGFß1 and TNFα and low proliferation of T lymphocytes stimulated polyclonally in-vitro. These findings suggest the persistence of an inflammatory immune response in patients with idiopathic RM that cannot be efficiently counter-regulated by up-regulated NK, NKT and T cell subsets with an immunoregulatory phenotype. The deficit of proliferation of T cells and TGFß1 production of NK cells after in-vitro stimulation might be a consequence of up-regulated immunosuppressive components of the immune system, increased consumption of cytokines in an autocrine manner by the activated immune system of the patients, or beginning exhaustion of pre-activated immune cells due to permanent activation of these particular cell subsets. These factors might partially explain the discrepancy between increased IL4+ and TGFß+ NK/NKT/T cells and decreased IL4 and TGFß levels in the plasma of iRM patients.

Although stable transplant recipients have normal counts of circulating IL4+ and TGFß+ NK, NKT and T cells, they have abnormally high IL4 and TGFß1 plasma levels suggesting that tissue resident NK, NKT and T cells and/or other cell subsets produced those cytokines. The higher plasma levels of these immunosuppressive cytokines might contribute to immunoregulatory immune mechanisms that allow the commonly practiced stepwise tapering of immunosuppressive drug doses in transplant recipients, eventually resulting in good long-term graft function in the presence of minimal doses of immunosuppressants. In contrast, iRM patients showed normal or slightly decreased IL4 and TGFß1 plasma levels. Yue et al. reported on significantly higher serum TGFß in the first trimester as compared to non-pregnant women whereas serum IL10 was similar in pregnant and non-pregnant women [13]. The same authors observed increased serum levels of immunosuppressive IL35 in normal early pregnancy and decreased levels in iRM patients [13]. Burns et al. reported on higher IL1β, IL6, IL8, TNFα, IFNγ, IL10, and IL1 receptor antagonist (IL-1RA) levels during late pregnancy in amniotic fluid than in cord blood or maternal plasma [14]. Decreased CCL5/RANTES plasma levels observed by us and others [15] may contribute to iRM pathogenesis because it was shown in-vitro that RANTES suppresses alloantigen specific T-cell responses [15] and promotes a pro-implantatory microenvironment that influences trophoblast cell survival and modulates the balance of maternal Treg/T effector lymphocytes in favor of maternal tolerance [16, 17]. Our data are in line with this observation and show low IL10, TGFß1, TNFα, IL1ß, IL1RA, IL5, IL6, IL12, GM-CSF, VEGF and CCL5 in the plasma of iRM patients, in agreement with the observations of Burns et al. [14]. A limitation of our study might be that a potential systemic increase of immunosuppressive cytokines was undetectable in our assays, possibly because the iRM patients were at least 2 months after the last pregnancy at the time of testing. The only cytokine that was highest in iRM patients compared to all other examined groups was G-CSF. Because G-CSF plays an effective role in pregnancy success [18] the strongly increased plasma levels may reflect a counter-regulation to overcome the pathomechanisms of iRM. Interestingly, G-CSF therapy is effective in reducing pregnancy loss [19].

It should be further mentioned that iRM patients showed higher Treg than female transplant recipients (*p* = 0.037) and similar Treg numbers as female healthy individuals (*p* = 0.933), as also shown previously [6]. IL10+ T cells were similar in all groups (iRM vs female transplant recipients *p* = 0.923 and vs female HC *p* = 0.486) [6].

Down-regulation of the stimulated immune system might be an option for balancing the immune system of iRM patients. In a small clinical trial, 40 patients with increased NK cells were treated with intralipid infusions that should exert an anti-inflammatory effect on the up-regulated immune system [20]. Our own preliminary data showed that iRM patients with very high pre-pregnancy peripheral NK lymphocyte counts did not benefit from lipid infusions and probably need additional (pre-pregnancy) medication [20]. In a recently published study, Liu et al. reported results of low-dose immunotherapy in iRM patients and showed lower proportions of TH1 cells and higher proportions of TH2 cells and Treg as well as higher TGFß1 serum levels after than before treatment [21]. Patients with immunotherapy achieved significantly more frequently pregnancy than untreated patients [21]. The role of immunotherapy in clinical practice is discussed controversially [22]. In consideration of live birth and abortion rates, treatment protocols using combinations of corticosteroid, low dose aspirin, unfractionated heparin, G-CSF, low molecular weight heparin and/or intravenous immunoglobulin appear to be effective treatment strategies [19]. Treatment with immunomodulatory substances should downregulate cytotoxic alloresponses and should induce immune mechanisms that establish a favorable micromilieu for the fetus.

Finally, from the perspective of a transplantation immunologist, which some of the authors are, female long-term transplant recipients and healthy females constitute interesting control groups for comparison with iRM patients. As detailed by Mor et al. [3], intervals with inflammatory and intervals with anti-inflammatory immune responses alternate during pregnancy. The first pregnancy trimester is shaped by an inflammatory, the second by an anti-inflammatory and the third again by an inflammatory immune response. These alterations are dynamic and there are phases in between showing both types of immune response. A control group of females with normal pregnancy would be rather heterogeneous with respect to the dominating immune response in the periphery depending on the time point of blood donation during pregnancy.

There is another aspect. According to our hypothesis, iRM patients had rejected the allogeneic fetus immunologically more than 3 times according to the definition of iRM. All iRM patients were investigated at least 3 months and maximally 12 months after the last abortion. One would think that the immune system of these female patients should be more comparable to the immune system of a non-pregnant than to that of a pregnant female. After abortion and antigen loss, the immune system of iRM patients should have returned to a resting state. However, as shown in this study and published in a previous manuscript [6], iRM patients showed an abnormally high cytotoxic NK cell response. We interpret this extremely high cytotoxic response as a long-term immune response against a persisting antigen and speculate that this cytotoxic immune response is directed towards fetal cells that persist in the iRM patients. Physiologically, immune responses stop when the triggering antigen is eliminated. Transplant recipients are facing a similar immunological challenge. They are continuously stimulated by an allograft. In contrast to the iRM patients, they show quiescence parameters of the immune system that are similar to female healthy individuals, as reported in our publications. Patients with good long-term graft function show normal cytotoxic and normal immunoregulatory responses in the circulation, which agrees with the notion that their immune system is in balance. The low maintenance doses of immunosuppressive drugs would not be sufficient to prevent acute graft rejection immediately post-transplant. Obviously, transplant recipients develop post-transplant immune mechanisms that facilitate the reduction of immunosuppressive drugs to a low maintenance level. The low maintenance dose of immunosuppressants is able to prevent rejection triggered by unspecific immune stimulation, induced for example by infections. Infections are able to trigger rejections by IFN increases and up-regulation of donor MHC antigens on cells of the transplant.

Female long-term transplant recipients and healthy females constitute interesting control groups for comparison with iRM patients. These control groups did not show the strong cytotoxic and counter-regulating immunoregulatory responses found in iRM patients, which supports the hypothesis that iRM patients, despite evidence for ongoing immunoregulatory activity, are not able to establish a low level balance of immunostimulatory (IFNy+ cells, plasma IFNy, TNFα, G-CSF, etc) and immunosuppressive (IL4+ and TGFß+ cells, plasma IL4, TGFß, IL10, etc.) mechanisms. We speculate that the missing balance prevents successful pregnancy and that immunosuppressive therapy might be an option to facilitate such a balance. Our conclusion is based on the observations obtained in female healthy controls and female long-term transplant recipients with good graft function. We feel that the observations in these individuals offer an interesting view on iRM patients that would not be obtained when iRM patients would be compared with normal pregnant women who are known to show heterogeneous immune reactions that depend on the duration of pregnancy, as described by Mor et al. [3].

## Conclusions

In summary, iRM patients show an activated immune system that can hardly be stimulated further and cannot be efficiently down-regulated by up-regulated TGFß1+ and IL4+ NK, NKT and T lymphocytes which are present concomitantly in these patients. The low IL4 and TGFß1 and strongly decreased IL10 plasma levels indicate deficient down-regulation and reflect a dysbalance of the immune system in iRM patients.

## Methods

### Patients and healthy controls

Healthy blood donors and laboratory staff served as controls. Blood samples from iRM patients were obtained during visits at the outpatient clinic of the Department of Obstetrics and Gynecology, University Hospital Heidelberg. Blood samples from transplant patients were obtained during regular visits in the outpatient clinics of the university hospitals in Heidelberg and Giessen. Table 1 summarizes the demographic patient data. Transplant recipients were treated with combinations of cyclosporine, tacrolimus, steroids and mycophenolate mofetil. iRM patients had a history of 3 or more consecutive miscarriages and anatomical disorders, endocrinologic dysfunctions, autoimmunologic disorders, deficiencies in coagulation factors, inherited haemostatic changes, and fetal and parental chromosomal disorders were excluded. Patients were investigated at least 2 months after the last miscarriage.

**Table 1:** Demographic data of patients and healthy controls

	HC (*n* = 35)	Late post-Tx (*n* = 37)	Early post-Tx (*n* = 10)	ESRD (*n* = 34)	iRM (*n* = 33)
Ages (years, median, SD)	44 (13)	55 (10)	58 (12)	52 (14)	34 (4)
Days post-transplant (median, range)	–	1602 (95–3786)	54 (7–93)	–	–
Sex (n, %)					
Female	19 (54%)	14 (38%)	3 (30%)	15 (44%)	33 (100%)
Male	16 (46%)	23 (62%)	7 (70%)	19 (56%)	0
End stage renal disease (n, %)					
Chronic glomerulonephritis	–	3 (8%)	2 (20%)	7 (21%)	–
Diabetes	–	3 (8%)	0	2 (6%)	–
Hypertension/ischemic	–	2 (5%)	1 (10%)	1 (3%)	–
Nephrosclerosis	–	2 (5%)	0	5 (15%)	–
Polycystic	–	10 (27%)	4 (40%)	7 (21%)	–
IgA nephropathy	–	6 (16%)	0	5 (15%)	–
Others	–	11 (30%)	3 (30%)	7 (21%)	–
Graft No. (n, %)					
First	–	35 (95%)	9 (90%)	–	–
Second	–	2 (5%)	1 (10%)	–	–
Donor (n, %)					
Living	–	26 (70%)	5 (50%)	–	–
Deceased	–	11 (30%)	5 (50%)	–	–
Cold ischemia time (n, %)					
< 3 h	–	26 (70%)	6 (60%)	–	–
> 3 h	–	11 (30%)	4 (40%)	–	–
Serum creatinine (n, %)					
< 2 mg/dl	–	33 (89%)	8 (80%)	–	–
> 2 mg/dl	–	4 (11%)	2 (20%)	34 (100%)	–
GFR (n, %)					
< 35 ml/min	–	3 (8%)	2 (20%)	34 (100%)	–
35–50 ml/min	–	12 (32%)	8 (80%)	–	–
> 50 ml/min	–	22 (60%)	0	–	–
HLA-ABDR mismatch > 3 (n, %)	–	11 (30%)	4 (40%)	–	–
CMV IgG+ recipients (n, %)	–	16 (43%)	8 (80%)	–	–
CMV IgG+ donors (n, %)	–	18 (49%)	8 (80%)	–	–

Abbreviations: *HC* healthy control, *late post-Tx* recipients late post-transplant, *early post-Tx* recipients early post-transplant, *ESRD* end stage renal disease, *iRM* idiopathic recurrent miscarriage

### Lymphocyte and NK cell subsets

Determination of lymphocyte and NK cell subsets was performed as previously described [6]. 150 μl of whole blood and fluorochrome-labeled monoclonal antibodies against CD45, CD16, CD56, CD3, and HLA-DR were added to each tube whereas IL4R, TGFßRII, IL10R, IFNyR and IgG-isotype controls were added only to certain tubes. Tubes were vortexed and incubated in the dark at room temperature for 30 min. Then, 2 ml of Lyse solution diluted 1:10 (BD Bioscience, Sunnyvale, CA, USA) was added and tubes were vortexed again, incubated at room temperature for 10 min and centrifuged at 1300 rpm for 8 min. After 2 washes of cells with 2 ml PBS, cells were suspended in 300 μl PBS and analyzed using a FACSCanto II triple-laser flow cytometer (BD Bioscience).

When intracellular proteins were analyzed, cells were permeabilized, in addition, by adding 500 μl of BD Perm/Wash buffer II diluted 1:10 (BD Bioscience). Cells were incubated for 10 min, 2 ml PBS was added, tubes were vortexed, centrifuged at 1300 rpm for 8 min, supernatant was removed and discarded and pellets were suspended in 100 μl PBS. Antibody against IL4, IL10, TGFß1 and IFNy was added and incubated for 30 min, tubes were vortexed and cells were washed twice in PBS. Samples were analyzed using eight-color fluorescence and a FACSCanto II triple-laser flow cytometer (BD Biosciences). At least 50,000 lymphocyte events were studied in the initial FSC/SSC dot plot (see gating strategy in Fig. 1). Because cells were not stimulated for intracellular staining of cytokines, our data reflect the cytokine production of NK, NKT and T cells in-vivo.

### Preparation of peripheral blood mononuclear cells and target cells before stimulation

Frozen PBMC were thawed as described previously [6]. Cell concentration was adjusted to 2 × 10^6^ cells/ml. Cells were stored overnight in an incubator at 37 °C and 5% CO_2_ atmosphere. K562 cell line was incubated at 37 °C and 5% CO_2_ and the culture medium was changed 24 h before the stimulation experiment.

### Six-hour multiple response assay

The multiple response assay was performed as described previously [6]. In brief, PBMC and K562 tumor cells were adjusted to 2 × 10^6^ cells/ml and 150 μl of PBMC were incubated with 30 μl of K562 tumor cells at 37 °C for 6 h using an E:T ratio of 5:1. After 1 h incubation time, 20 μl of cell culture medium supplemented with Monensin (Golgistop, BD Bioscience) diluted 1:100 was added. Then cells were incubated for 5 h, centrifuged at 300 g for 5 min, suspended in 100 μl PBS, stained with fluorochrome-labeled monoclonal antibody CD3, CD56, CD16, CD45, and HLA-DR and incubated for 30 min at room temperature in the dark. Cells were washed and permeabilized using BD Perm/Wash buffer II (BD Bioscience). Monoclonal antibody against TGFß1, IL4, IL10 or IFNy were added, samples were incubated for 30 min at room temperature in the dark, washed with permeabilization washing buffer and suspended in 300 μl PBS. Fluorescence of cells was analyzed using an eight-color fluorescence flow cytometer FACS Canto II (BD Biosciences).

### Determination of cytokines and chemokines in plasma and supernatants

IL-1β, IL-2, IL-4, IL-5, IL-6, IL-8, IL-10, IL-12 p70, GM-CSF, IFN-γ, TNF-α and VEGF (Luminex Performance Assay, Human High Sensitivity Cytokine Base Kit A; R&D systems, Wiesbaden, Germany), CCL2/MCP-1, CCL3/MIP-1α, CCL4/MIP-1ß, CCL5/Rantes, CXCL5/ENA-78, FGF basic, G-CSF and Thrombopoietin/TPO (Human Luminex Performance Assay Base Kit, Panel A; R&D systems, Wiesbaden, Germany) and TGFß1, TGFß2 and TGFß3 (Luminex Performance Assay 3-plex Kit; R&D systems, Wiesbaden, Germany) were determined in plasma and supernatants according to instructions of the manufacturer and analyzed using the Luminex LX100 system (Luminex B.V., Het Zuiderkruis 1, 5215 MV ‘s-Hertogenbosch, The Netherlands).

### Statistical analysis

PASW Statistics program version 21 (IBM, Chicago, Illinois, USA), Wilcoxon signed rank test and Mann-Whitney U test were used for statistical analysis. With respect of the interpretation of the test results, lymphocyte subsets were devided into cells with either immunostimulatory (IFNy+, etc.) or immunoregulatory phenotype (IL4+, IL10+, TGFß+, etc.) showing a trend whether the immune system is stimulated or immunosuppressed. Therefore, we did not adapt *p*-values according to Bonferroni correction and considered a result with a p-value of < 0.050 as significant.

## Additional files

Additional file 1: **Figure S1a + b.** IL4+, TGFß+, IL10+ and IFNy+ NK, NKT and T cell counts in peripheral blood. iRM patients showed higher absolute numbers of circulating NK, NKT and T lymphocytes producing IL4, TGFß1 and IFNy than male and female HC, ESRD and transplant patients late post-transplant (for all *p* < 0.050) with the exception of IFNy+ NK cells which were similar in iRM patients and male and female kidney transplant recipients early post-transplant (p = n.s.). Absolute numbers of IL10+ NK, NKT and T cells were similar in iRM patients and male and female HC. Thirty-five HC, 34 ESRD, 37 renal transplant recipients late and 10 renal transplant recipients early post-transplant as well as 33 iRM patients were studied. Data are given as median ± interquartile range. (ZIP 1402 kb)

Additional file 2: **Figure S2.** IL4R+, TGFßR+, IFNyR+ and IL10R+ NK, NKT and T cell counts in peripheral blood. NK cells of iRM patients showed lower TGFßR expression than those of male and female transplant recipients early and late post-transplant (for all p < 0.050). iRM showed the highest absolute count of IFNyR+ NK cells of all examined groups (for all *p* < 0.050), suggesting a low need for paracrine produced TGFß and a strong response of IFNyR+ NK cells. Thirty-five HC, 34 ESRD, 37 renal transplant recipients late post-transplant and 10 renal transplant recipients early post-transplant as well as 33 iRM patients were studied. Data are given as median ± interquartile range. (DOCX 1032 kb)

Additional file 3: **Figure S3a + b.** Plasma levels of cytokines and chemokines. iRM patients showed the lowest plasma levels of TGFß1, TGFß3, IL10, TNFα, IL1ß, IL6, IL8, IL17 and GM-CSF and the highest plasma levels of G-CSF and CXCL5 of all examined groups (for all p < 0.050). Moreover, they had normal levels of IL2, IL4, IL12, TPO and VEGF that were lower than those in male and female ESRD and transplant recipients early and late post-transplant (for all p < 0.050). IFNy, IL1α and TGFß2 plasma levels were similar in iRM patients, male and female HC and transplant recipients (for all p > 0.050). Thirty-five HC, 34 ESRD, 37 renal transplant recipients late and 10 renal transplant recipients early post-transplant as well as 33 iRM patients were studied. Data are given as median ± interquartile range. (ZIP 2437 kb)

## Abbreviations

ESRD: End stage renal disease
FSC: Forward-scattered light
GFR: Glomerular filtration rate
HC: Healthy control
iRM: Idiopathic recurrent miscarriage
MTHFR: Methylenetetrahydrofolate reductase
PBL: Peripheral blood lymphocytes
PBMC: Peripheral blood mononuclear cells
PBS: Phosphate buffered saline
SSC: Side-scattered light
TX: Transplant recipient
