# Supplementary figures and images for: Patients with idiopathic recurrent miscarriage have abnormally high TGFß+ blood NK, NKT and T cells in the presence of abnormally low TGFß plasma levels
Source: BMC Immunol. 2019 Mar 4;20:10. doi: 10.1186/s12865-019-0290-3 (PMC6399890; doi:10.1186/s12865-019-0290-3)

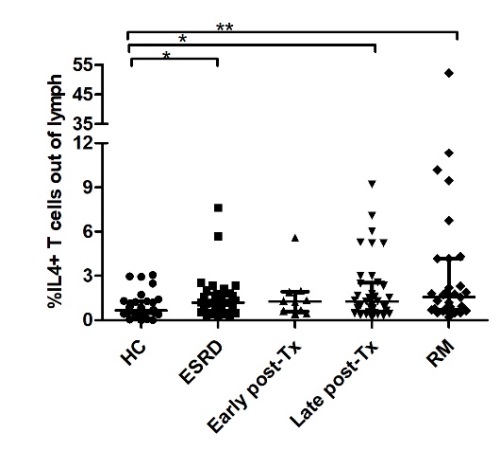

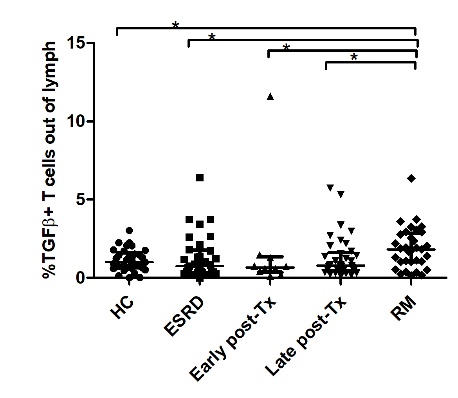

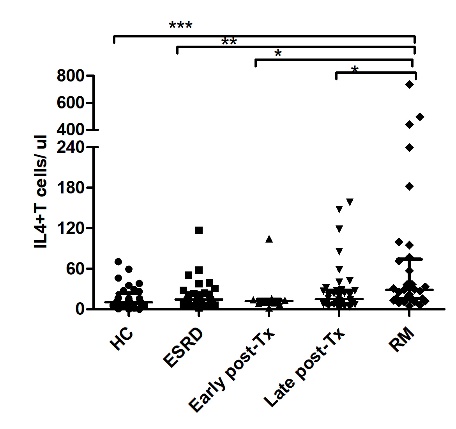

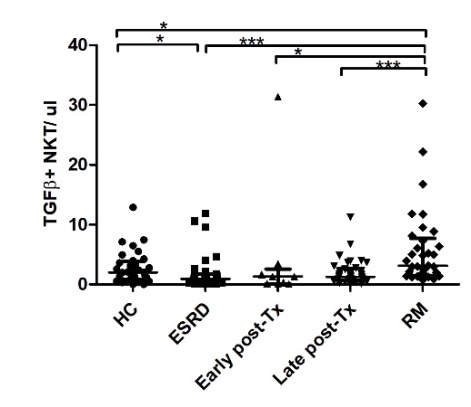

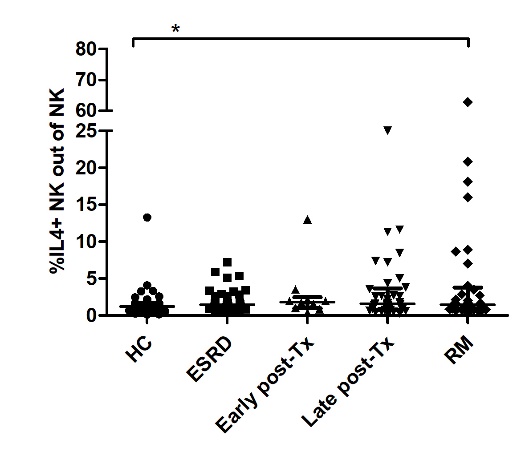

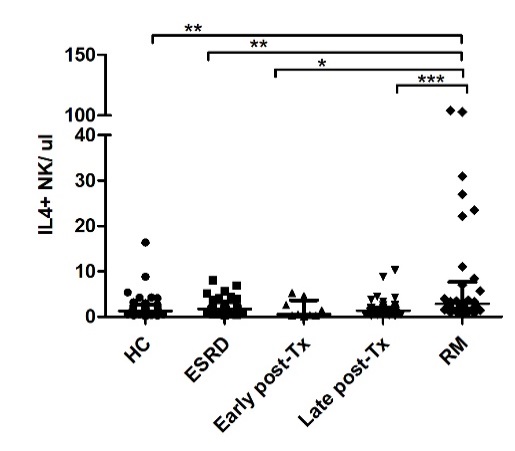

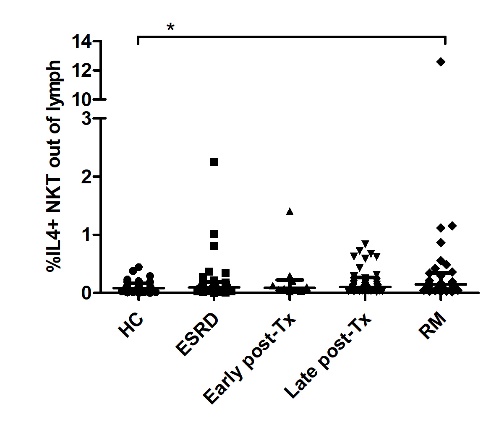

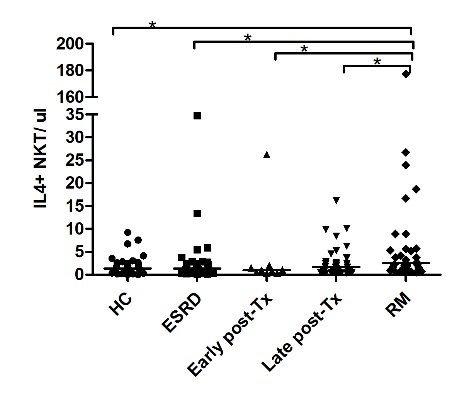

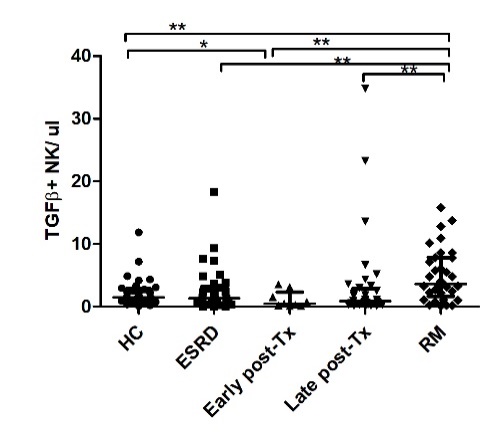

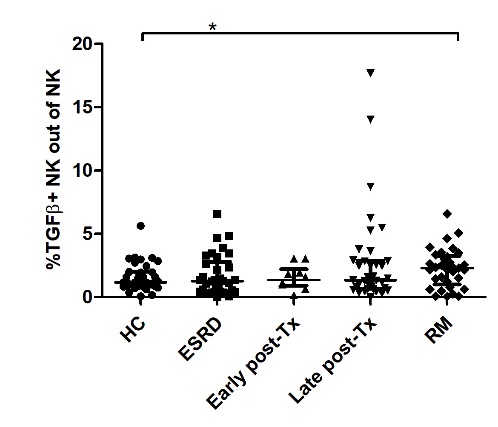

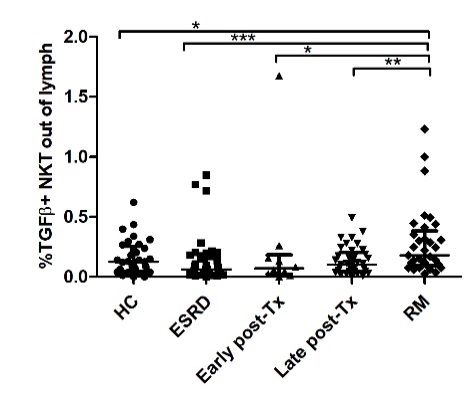

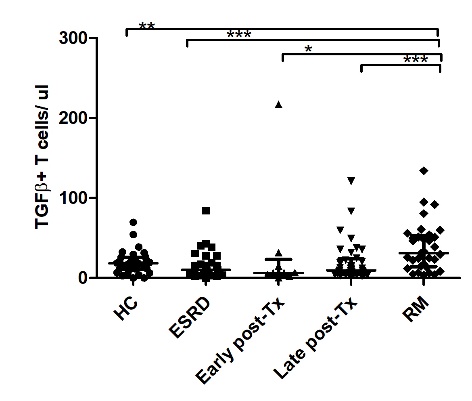


**Figure 1a, Supplementary file**

**A**

**B**

Supplement: Supplementary file 1 — Figure S1a + b. IL4+, TGFß+, IL10+ and IFNy+ NK, NKT and T cell counts in peripheral blood. iRM patients showed higher absolute numbers of circulating NK, NKT and T lymphocytes producing IL4, TGFß1 and IFNy than male and female HC, ESRD and transplant patients late post-transplant (for all p < 0.050) with the exception of IFNy+ NK cells which were similar in iRM patients and male and female kidney transplant recipients early post-transplant (p = n.s.). Absolute numbers of IL10+ NK, NKT and T cells were similar in iRM patients and male and female HC. Thirty-five HC, 34 ESRD, 37 renal transplant recipients late and 10 renal transplant recipients early post-transplant as well as 33 iRM patients were studied. Data are given as median ± interquartile range. (ZIP 1402 kb) [file 12865_2019_290_MOESM1_ESM.zip › Plasma cytokines 32 Supplementary Figure 1a rev.docx]

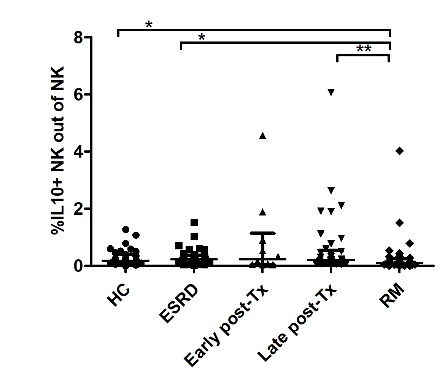

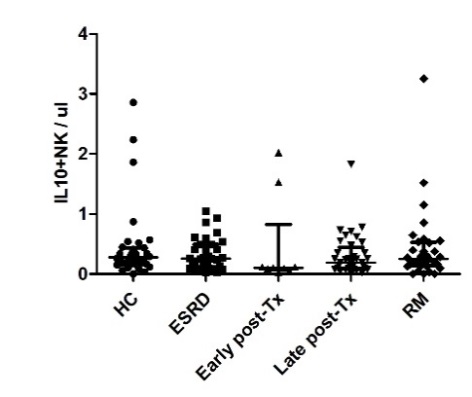


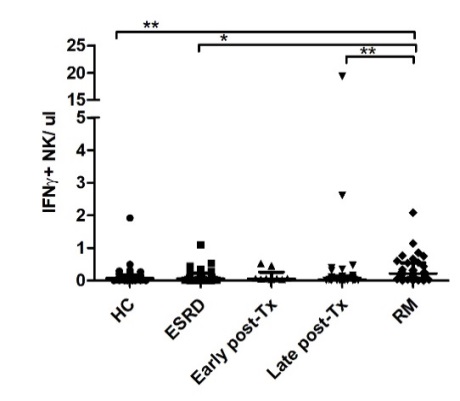

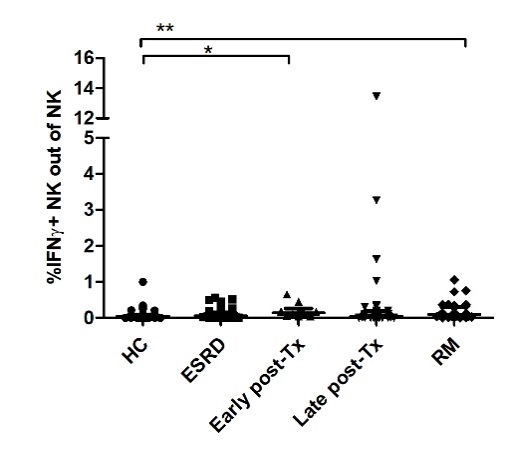

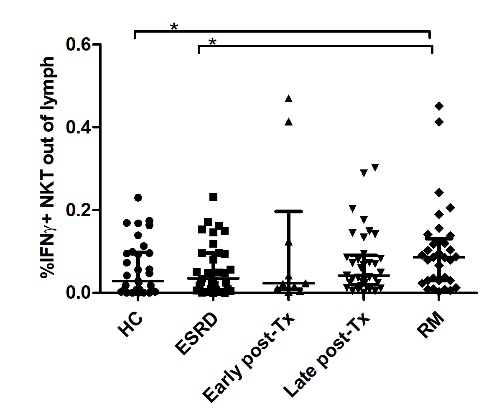

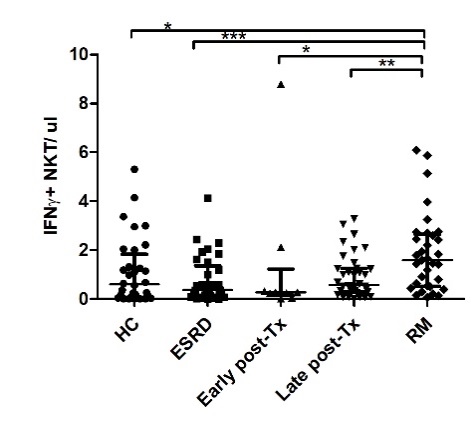

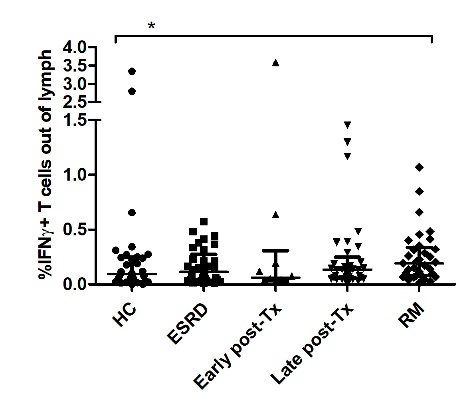

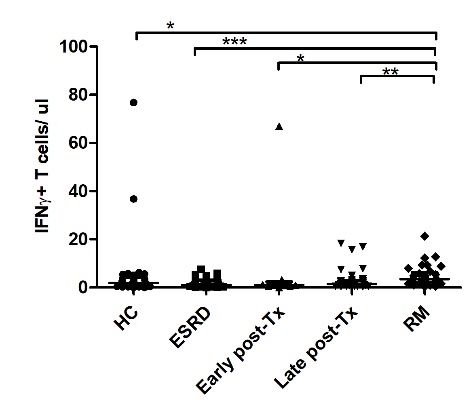


**D**


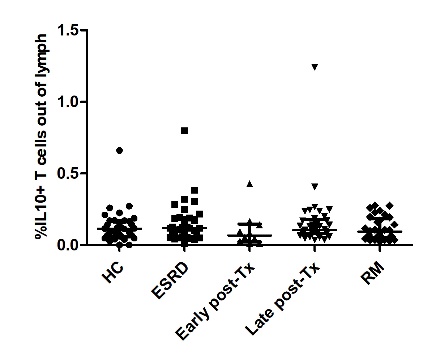


**C**


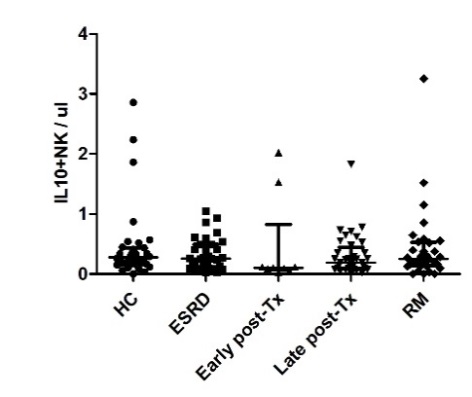

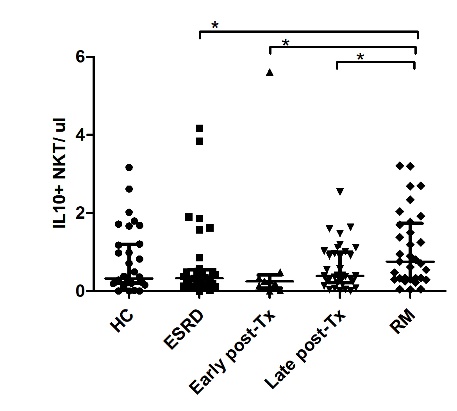

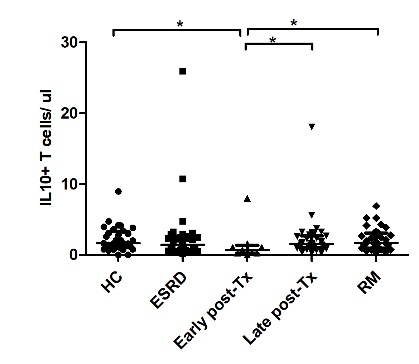

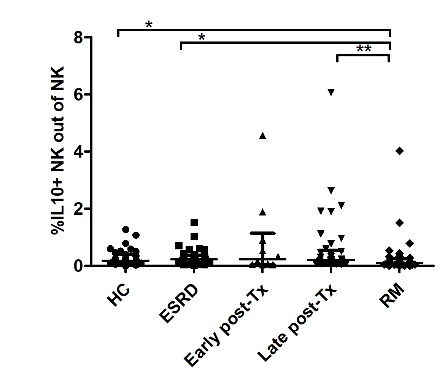

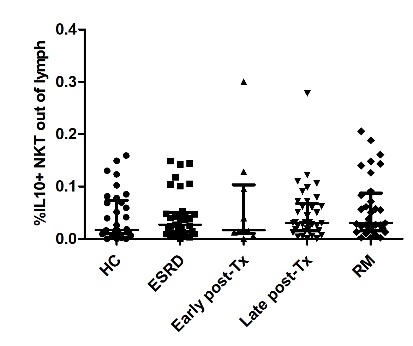


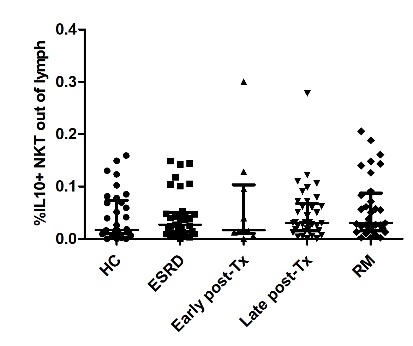

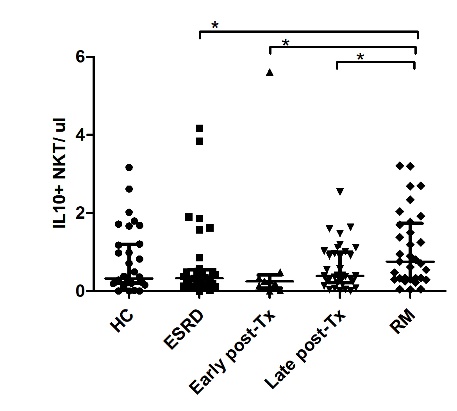


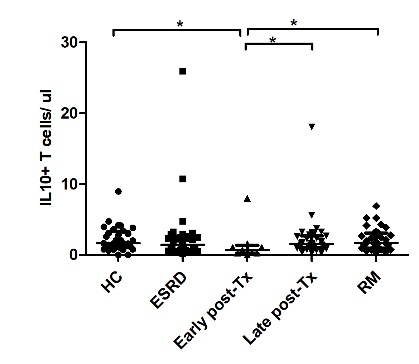


**Figure 1b, Supplementary file**

Supplement: Supplementary file 1 — Figure S1a + b. IL4+, TGFß+, IL10+ and IFNy+ NK, NKT and T cell counts in peripheral blood. iRM patients showed higher absolute numbers of circulating NK, NKT and T lymphocytes producing IL4, TGFß1 and IFNy than male and female HC, ESRD and transplant patients late post-transplant (for all p < 0.050) with the exception of IFNy+ NK cells which were similar in iRM patients and male and female kidney transplant recipients early post-transplant (p = n.s.). Absolute numbers of IL10+ NK, NKT and T cells were similar in iRM patients and male and female HC. Thirty-five HC, 34 ESRD, 37 renal transplant recipients late and 10 renal transplant recipients early post-transplant as well as 33 iRM patients were studied. Data are given as median ± interquartile range. (ZIP 1402 kb) [file 12865_2019_290_MOESM1_ESM.zip › Plasma cytokines 32 Supplementary Figure 1b rev.docx]

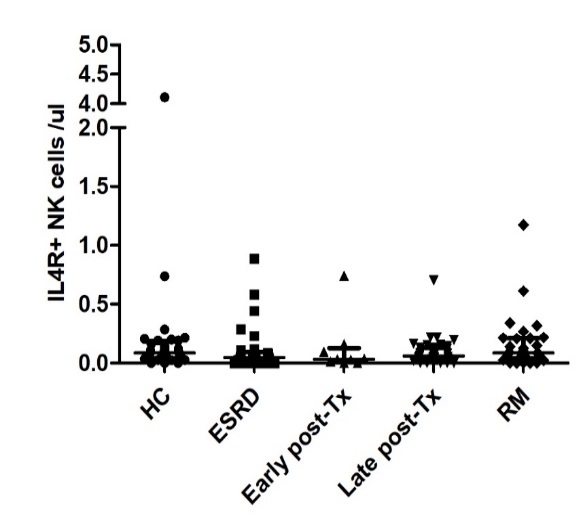

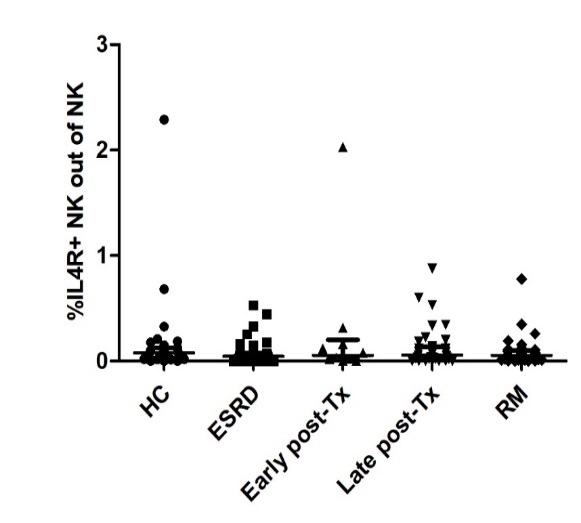


**A**


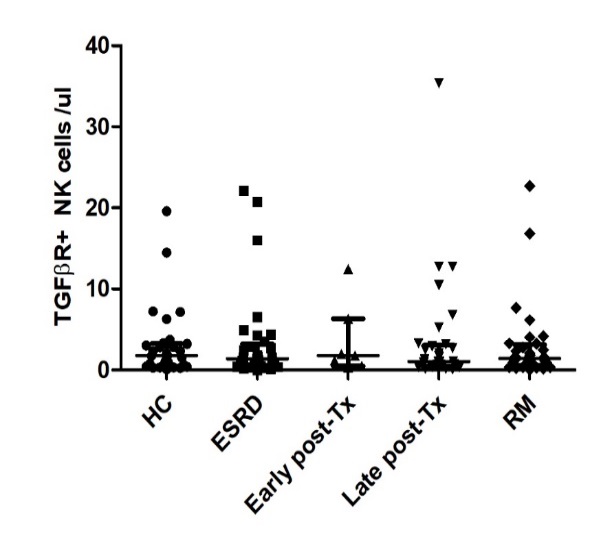

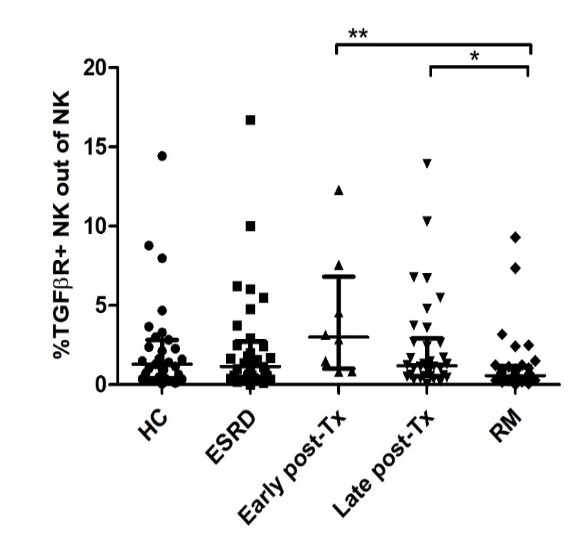


**B**


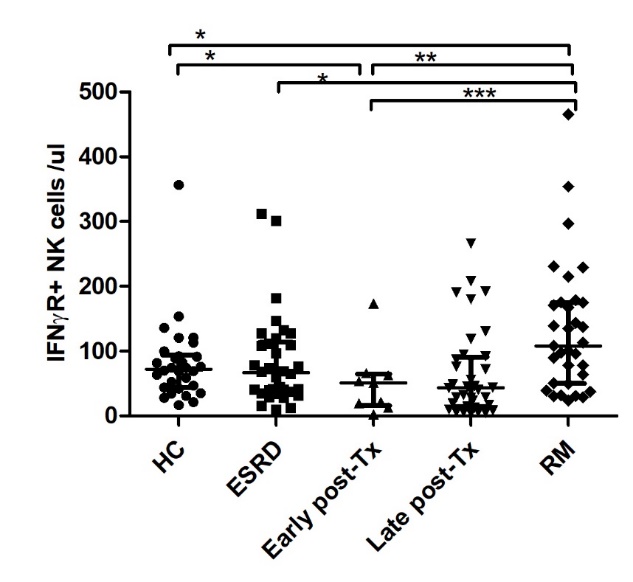

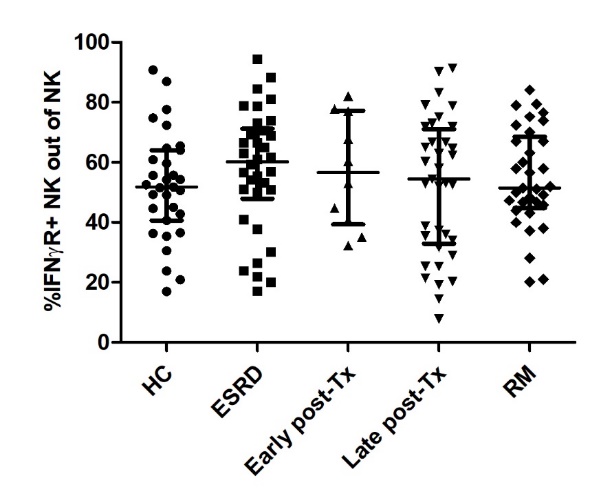


**C**


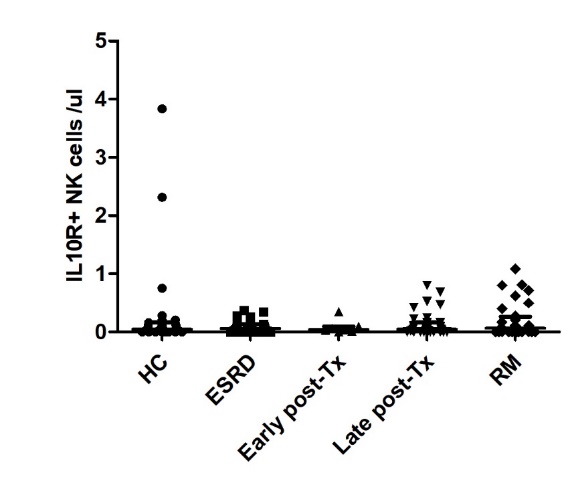

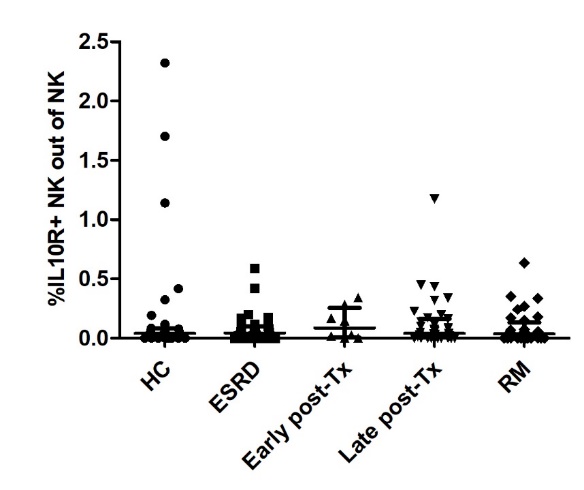


**D**

**Figure 2, Supplementary file**

Supplement: Supplementary file 2 — Figure S2. IL4R+, TGFßR+, IFNyR+ and IL10R+ NK, NKT and T cell counts in peripheral blood. NK cells of iRM patients showed lower TGFßR expression than those of male and female transplant recipients early and late post-transplant (for all p < 0.050). iRM showed the highest absolute count of IFNyR+ NK cells of all examined groups (for all p < 0.050), suggesting a low need for paracrine produced TGFß and a strong response of IFNyR+ NK cells. Thirty-five HC, 34 ESRD, 37 renal transplant recipients late post-transplant and 10 renal transplant recipients early post-transplant as well as 33 iRM patients were studied. Data are given as median ± interquartile range. (DOCX 1032 kb) [file 12865_2019_290_MOESM2_ESM.docx]

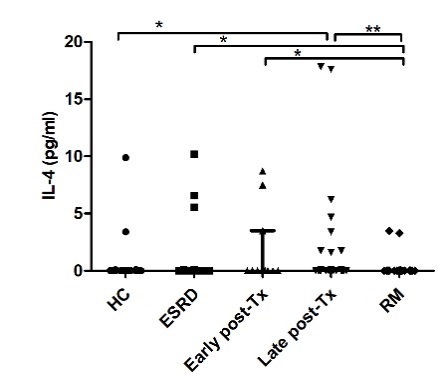

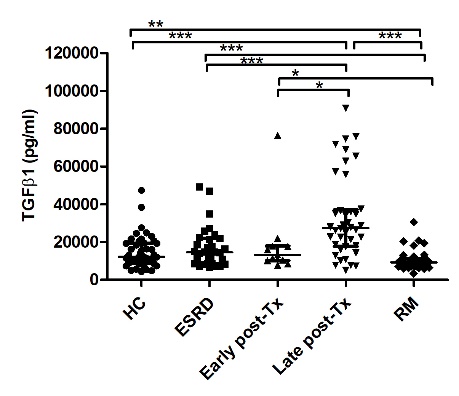

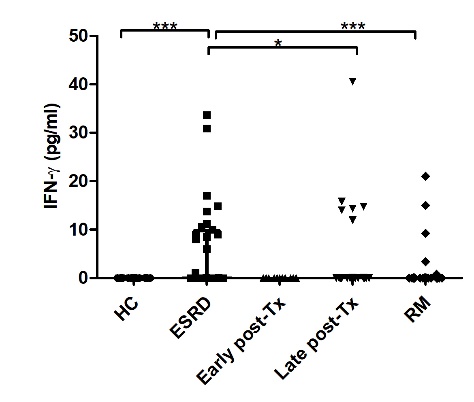

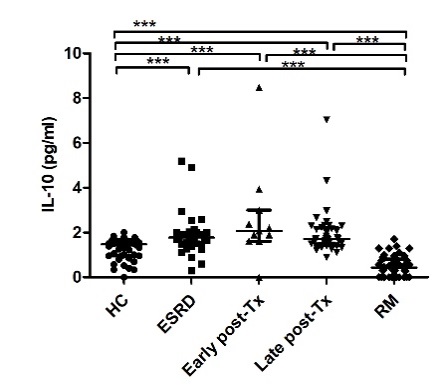

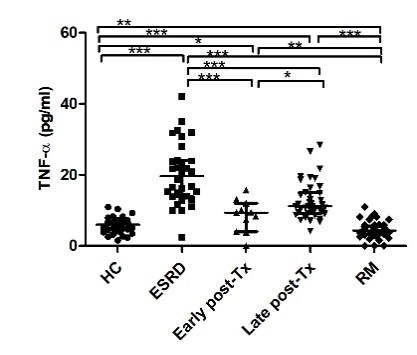

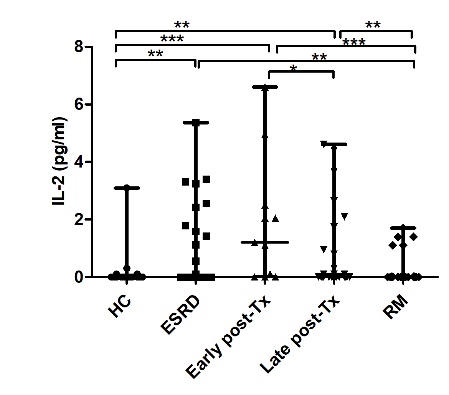

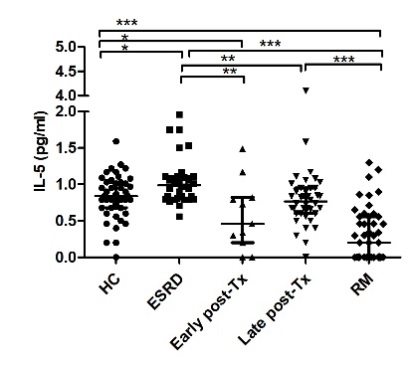

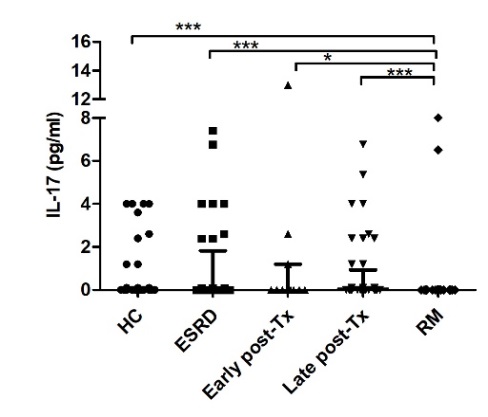

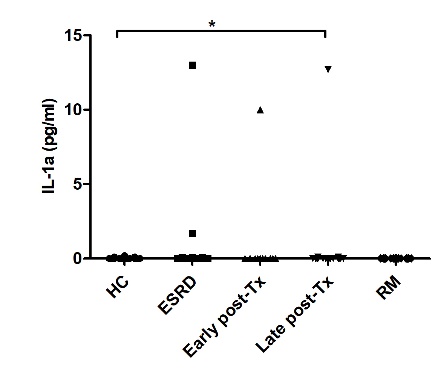

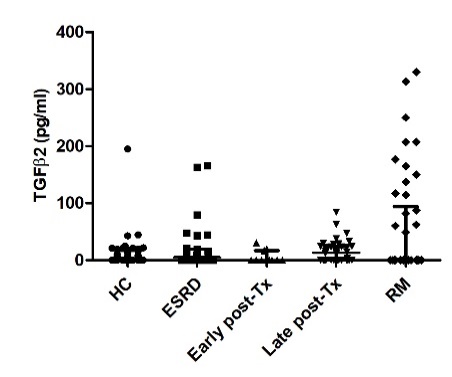

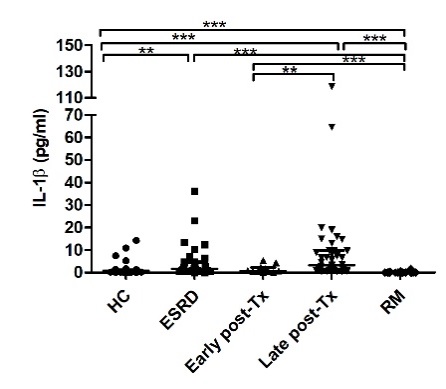

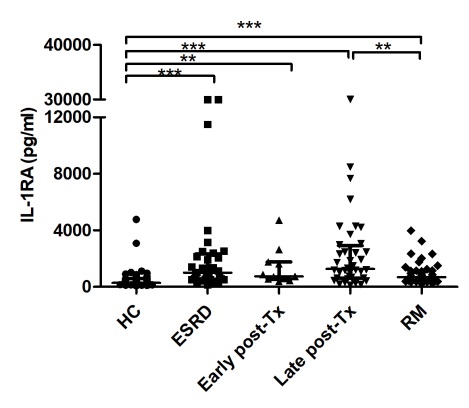

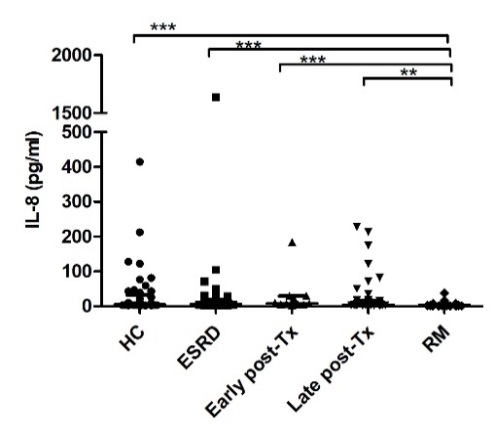

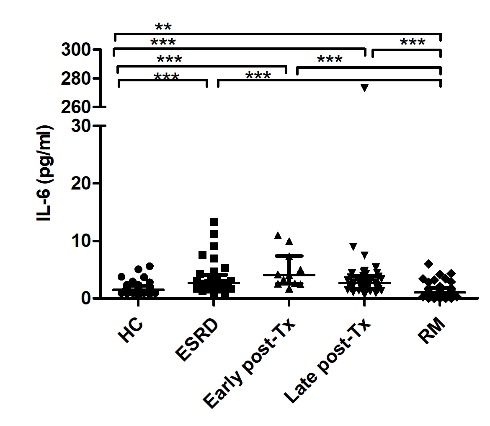

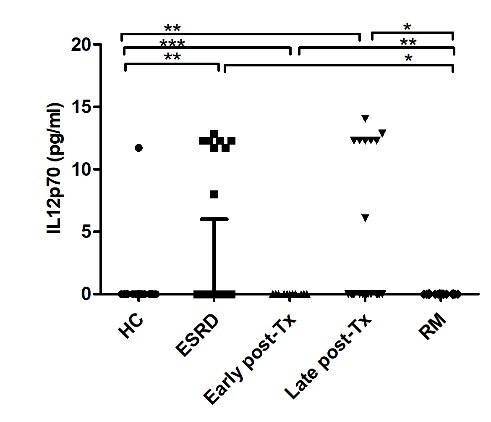


**Figure 3a, Supplementary file**

Supplement: Supplementary file 3 — Figure S3a + b. Plasma levels of cytokines and chemokines. iRM patients showed the lowest plasma levels of TGFß1, TGFß3, IL10, TNFα, IL1ß, IL6, IL8, IL17 and GM-CSF and the highest plasma levels of G-CSF and CXCL5 of all examined groups (for all p < 0.050). Moreover, they had normal levels of IL2, IL4, IL12, TPO and VEGF that were lower than those in male and female ESRD and transplant recipients early and late post-transplant (for all p < 0.050). IFNy, IL1α and TGFß2 plasma levels were similar in iRM patients, male and female HC and transplant recipients (for all p > 0.050). Thirty-five HC, 34 ESRD, 37 renal transplant recipients late and 10 renal transplant recipients early post-transplant as well as 33 iRM patients were studied. Data are given as median ± interquartile range. (ZIP 2437 kb) [file 12865_2019_290_MOESM3_ESM.zip › Plasma cytokines 32 Supplementary Figure 3a rev.docx]

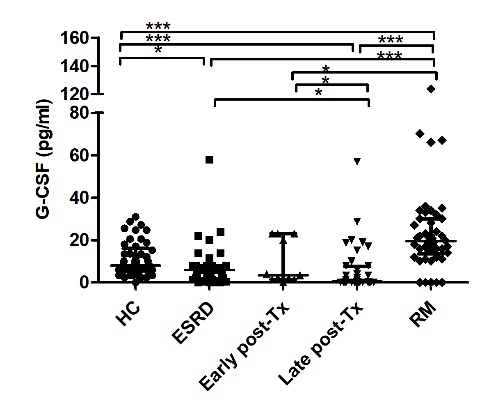

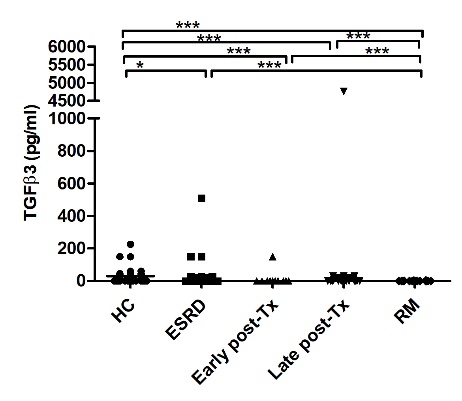

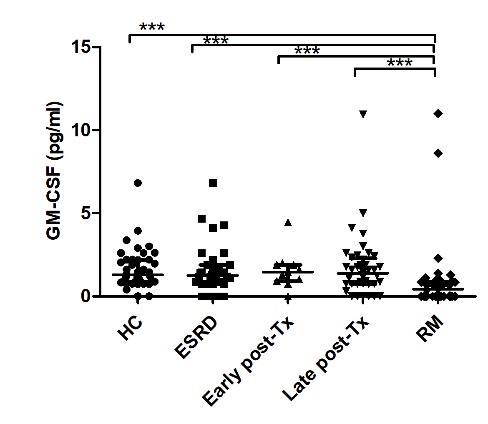

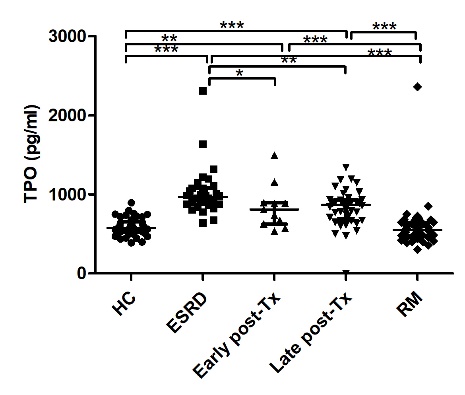

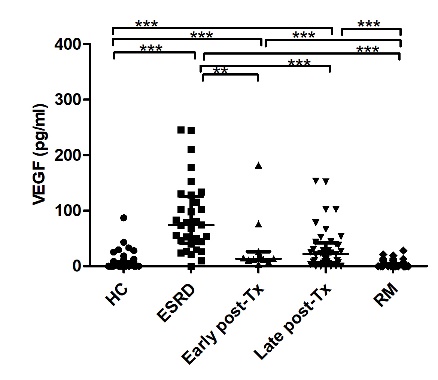

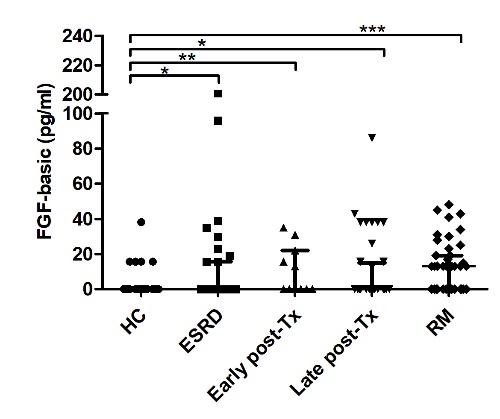

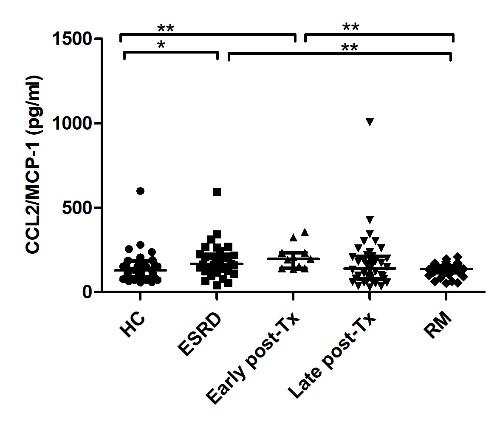

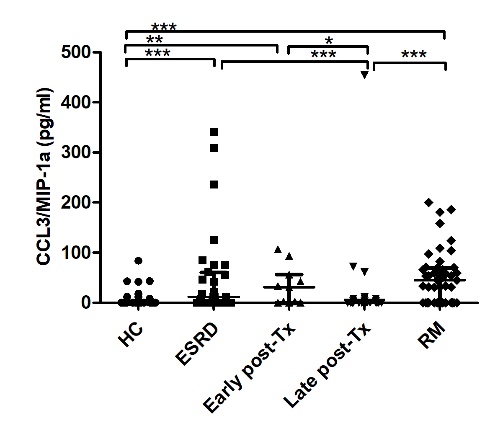

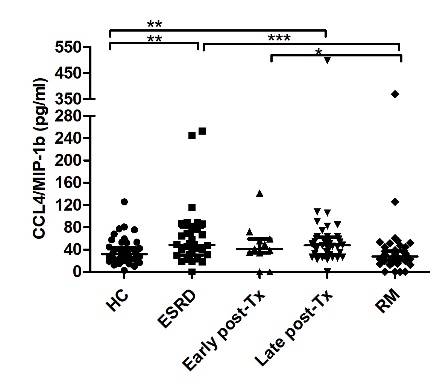

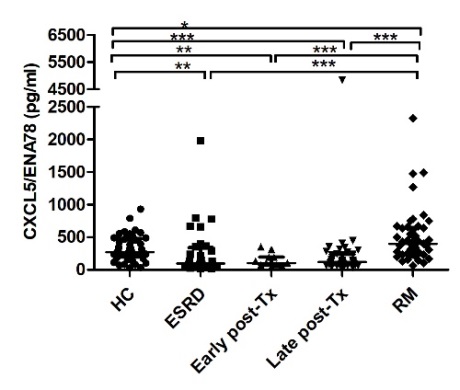

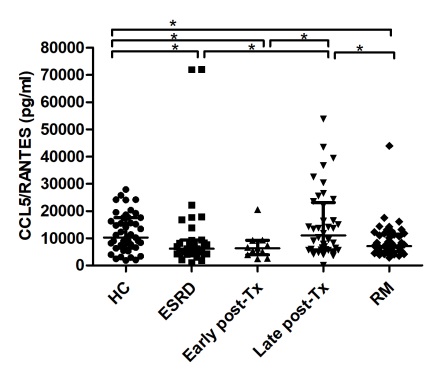


**Figure 3b, Supplementary file**

Supplement: Supplementary file 3 — Figure S3a + b. Plasma levels of cytokines and chemokines. iRM patients showed the lowest plasma levels of TGFß1, TGFß3, IL10, TNFα, IL1ß, IL6, IL8, IL17 and GM-CSF and the highest plasma levels of G-CSF and CXCL5 of all examined groups (for all p < 0.050). Moreover, they had normal levels of IL2, IL4, IL12, TPO and VEGF that were lower than those in male and female ESRD and transplant recipients early and late post-transplant (for all p < 0.050). IFNy, IL1α and TGFß2 plasma levels were similar in iRM patients, male and female HC and transplant recipients (for all p > 0.050). Thirty-five HC, 34 ESRD, 37 renal transplant recipients late and 10 renal transplant recipients early post-transplant as well as 33 iRM patients were studied. Data are given as median ± interquartile range. (ZIP 2437 kb) [file 12865_2019_290_MOESM3_ESM.zip › Plasma cytokines 32 Supplementary Figure 3b rev.docx]
